# Aminosugar-based immunomodulator lipid A: synthetic approaches

**DOI:** 10.3762/bjoc.14.3

**Published:** 2018-01-04

**Authors:** Alla Zamyatina

**Affiliations:** 1 Department of Chemistry, University of Natural Resources and Life Sciences, Muthgasse 18, 1190 Vienna, Austria

**Keywords:** glycoconjugate, glycolipids, glycosylation, immunomodulation, lipopolysaccharide, TLR4

## Abstract

The immediate immune response to infection by Gram-negative bacteria depends on the structure of a lipopolysaccharide (LPS, also known as endotoxin), a complex glycolipid constituting the outer leaflet of the bacterial outer membrane. Recognition of picomolar quantities of pathogenic LPS by the germ-line encoded Toll-like Receptor 4 (TLR4) complex triggers the intracellular pro-inflammatory signaling cascade leading to the expression of cytokines, chemokines, prostaglandins and reactive oxygen species which manifest an acute inflammatory response to infection. The “endotoxic principle” of LPS resides in its amphiphilic membrane-bound fragment glycophospholipid lipid A which directly binds to the TLR4·MD-2 receptor complex. The lipid A content of LPS comprises a complex mixture of structural homologs varying in the acylation pattern, the length of the (*R*)-3-hydroxyacyl- and (*R*)-3-acyloxyacyl long-chain residues and in the phosphorylation status of the β(1→6)-linked diglucosamine backbone. The structural heterogeneity of the lipid A isolates obtained from bacterial cultures as well as possible contamination with other pro-inflammatory bacterial components makes it difficult to obtain unambiguous immunobiological data correlating specific structural features of lipid A with its endotoxic activity. Advanced understanding of the therapeutic significance of the TLR4-mediated modulation of the innate immune signaling and the central role of lipid A in the recognition of LPS by the innate immune system has led to a demand for well-defined materials for biological studies. Since effective synthetic chemistry is a prerequisite for the availability of homogeneous structurally distinct lipid A, the development of divergent and reproducible approaches for the synthesis of various types of lipid A has become a subject of considerable importance. This review focuses on recent advances in synthetic methodologies toward LPS substructures comprising lipid A and describes the synthesis and immunobiological properties of representative lipid A variants corresponding to different bacterial species. The main criteria for the choice of orthogonal protecting groups for hydroxyl and amino functions of synthetically assembled β(1→6)-linked diglucosamine backbone of lipid A which allows for a stepwise introduction of multiple functional groups into the molecule are discussed. Thorough consideration is also given to the synthesis of 1,1′-glycosyl phosphodiesters comprising partial structures of 4-amino-4-deoxy-β-L-arabinose modified *Burkholderia* lipid A and galactosamine-modified *Francisell*a lipid A. Particular emphasis is put on the stereoselective construction of binary glycosyl phosphodiester fragments connecting the anomeric centers of two aminosugars as well as on the advanced P(III)-phosphorus chemistry behind the assembly of zwitterionic double glycosyl phosphodiesters.

## Introduction

**Figure 1:**
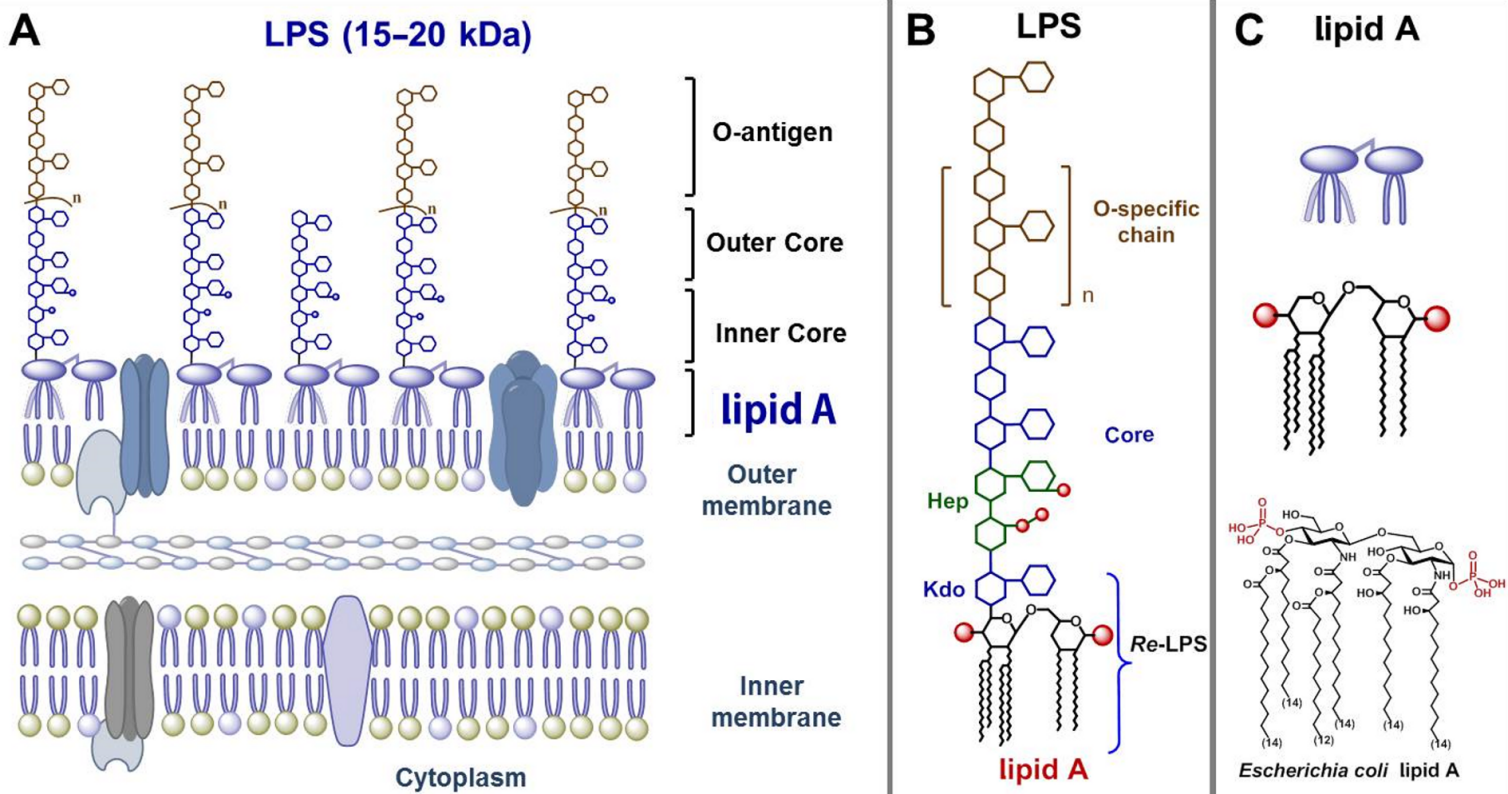
(A) Gram-negative bacterial membrane with LPS as major component of the outer membrane; (B) structural constituents of LPS: lipid A, inner/outer core and O-specific chain.

The mammalian innate immune system possesses an efficient and incredibly complex evolutionary ancient machinery responsible for host defence against pathogens. The receptors of the innate immune system can detect particular components present in bacteria, viruses or fungi which are designated as “pathogen associated molecular patterns” (PAMPs) [1]. These receptors, termed pattern recognition receptors (PRRs), are able of sensing and responding to PAMPs. The major surface antigen of Gram-negative bacteria, a complex heterogeneous glycolipid lipopolysaccharide (LPS, or endotoxin) [2–3], is recognised by a receptor complex composed of Toll-like Receptor 4 (TLR4) and a co-receptor protein myeloid differentiation factor 2 (MD-2) which are expressed by mammalian immune cells such as macrophages, monocytes and dendritic cells [4]. LPS represents the major virulence factor of Gram-negative bacteria and is essential for bacterial survival. LPS constitutes the outer leaflet of the outer membrane of Gram-negative bacteria (Figure 1A) and possesses a complex micro-heterogeneous structure distinguished by three regions: the lipid A [5], the core oligosaccharide [6] and the O-antigen [7] (Figure 1B). The TLR4·MD-2 receptor complex senses picomolar amounts of LPS and initiates the biosynthesis of diverse mediators of inflammation (such as tumor necrosis factor-TNF-α, interleukin 6 (IL-6) and IL-8) thereby triggering a downstream pro-inflammatory signaling cascade aimed at the clearance of infection [8]. Thus, LPS-induced TLR4·MD-2-mediated signaling largely contributes to the development of inflammation and initiation of the beneficial defensive host response which is essential for bacterial clearance and managing the Gram-negative bacterial disease.

However, under circumstances of an upregulated inflammation, the TLR4 activation results in the excessive production of the pro-inflammatory mediators [9] leading to overstimulation of the innate immune system and systemic inflammatory response syndrome (SIRS) which eventually results in a life-threatening sepsis syndrome and lethal septic shock [10–11] (the 10th leading cause of death in developed countries, 40–60% mortality rate) [12–13]. The membrane-bound portion of LPS, a glycophospholipid lipid A (Figure 1C), constitutes the “endotoxic principle” of LPS [14–15]. In depth studies demonstrated that the lipid A moiety of *E. coli* LPS causes a similar scope of sepsis-associated effects as its parent LPS which confirmed the proposed key role of lipid A in Gram-negative sepsis syndrome [15].

**Figure 2:**
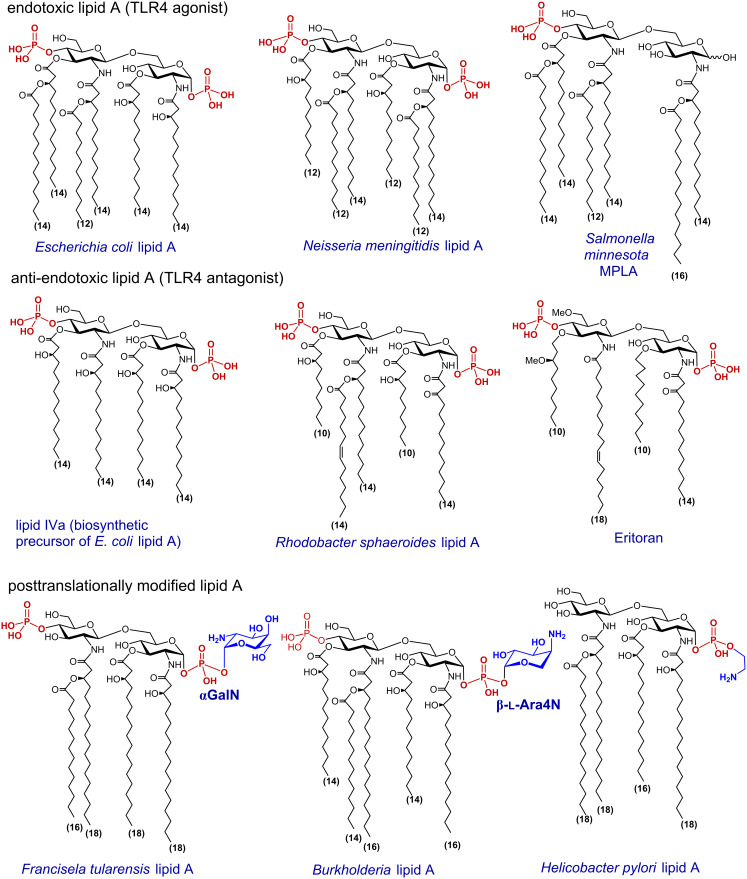
Structures of representative TLR4 ligands: TLR4 agonists (*E. col*i lipid A, *N. meningitidis* lipid A and MPLA) and TLR4 antagonists (lipid IVa, *R. sphaeroides* lipid A and eritoran (E5564)); examples of post-translationally modified lipid A from *Francisella*, *Burkholderia* and *Helicobacter*.

The chemical structure of lipid A is based on the β(1→6)-linked 1-,4′-bisphosphorylated diglucosamine backbone which is typically tetra- till heptaacylated at the amino groups (positions 2 and 2’) and hydroxyl groups (positions 3 and 3’) by (*R*)-3-hydroxy- or/and (*R*)-3-acyloxyacyl fatty acids of variable lengths usually comprising 12–16 carbon atoms [16–17]. The endotoxic activity of lipid A depends on numerous factors such as acylation and phosphorylation pattern [18], the length of lipid chains, and the tertiary 3D structure of the MD-2 bound βGlcN(1→6)GlcN backbone [19–20]. The most profoundly studied lipid A of *Escherichia coli* and *Neisseria meningitidis* contains six acyl chains (C_14_–C_12_) differently distributed across the diglucosamine backbone and two phosphate groups – one at the anomeric position of the proximal GlcN residue and the second at position 4’ of the distal GlcN moiety (Figure 2). These lipid A variants are highly endotoxic and represent the most effective stimulators of the intracellular pro-inflammatory signaling. However, partial activation of the TLR4·MD-2 complex by certain lipid A substructures (such as 1-*O*-dephosphorylated *Salmonella minnesota* lipid A – a licenced vaccine adjuvant monophosphoryl lipid A, MPLA – leads to the induction of a different cytokine profile that weakens toxicity but preserves the beneficial adjuvant effects of endotoxin. Other Gram-negative bacteria can produce lipid A variants which are either less endotoxic or inactive (e.g., cannot be recognised by the TLR4∙MD-2 complex) such as tetraacylated 1-*O*-monophosphorylated *Helicobacter pylori* lipid A (Figure 2) [21]. Underacylated lipid A of some Gram-negative organisms exhibit TLR4 antagonist activity, for example, pentaacyl lipid A from *Rhodobacter sphaeroides* [22] or C_14_-tetraacylated biosynthetic precursor of *E. coli* lipid A, lipid IVa [23] (Figure 2).

Many Gram-negative bacteria, particularly those with mammalian and environmental reservoirs, can produce modified forms of LPS in response to growth conditions, especially in response to a shift in growth temperature (e.g, 37 °C in human host vs 25 °C in a disease vector). These modifications include, in the first line, a cleavage of one or more acyl chains from the lipid A portion of LPS which results in the production of underacylated LPS variants which are “overseen” by the innate immune system of the host. For instance, *Yersinia pestis* produces tetraacylated lipid A in mammalian host compared to the hexaacylated lipid A in the insect vector which renders the bacterium resistant to the hosts innate immune system [24]. Lipid A modifications result in the “remodeling” of the bacterial membrane which alters the outer membrane integrity and antigen presentation, decreases susceptibility to antimicrobial peptides and enhances pathogenicity [25]. In some LPS, the lipid A phosphates are post-translationally modified by substitution with the compounds that reduce the net negative charge of LPS, such as phosphoethanolamine in *E. coli* and *Salmonella* [2,26], ethanolamine in *Helicobacter pylori*, 4-amino-4-deoxy-β-L-arabinose (β-L-Ara4N) [27–28] in *E. coli* [29], *Burkholderia* [27] and *Yersinia pestis* [30] or galactosamine in *Francisella* [2,26], and glucosamine in *Bordetella* species [31] (Figure 2). Covalent attachment of aminosugar to the phosphate groups of lipid A alters the TLR4-mediated host immunity and accounts for the modulation of the pro-inflammatory signaling. Additionally, this modification is associated with an amplified bacterial virulence since it confers resistance to the endogenous cationic antimicrobial peptides (CAMPs) and antibiotics [25,32–34].

**Figure 3:**
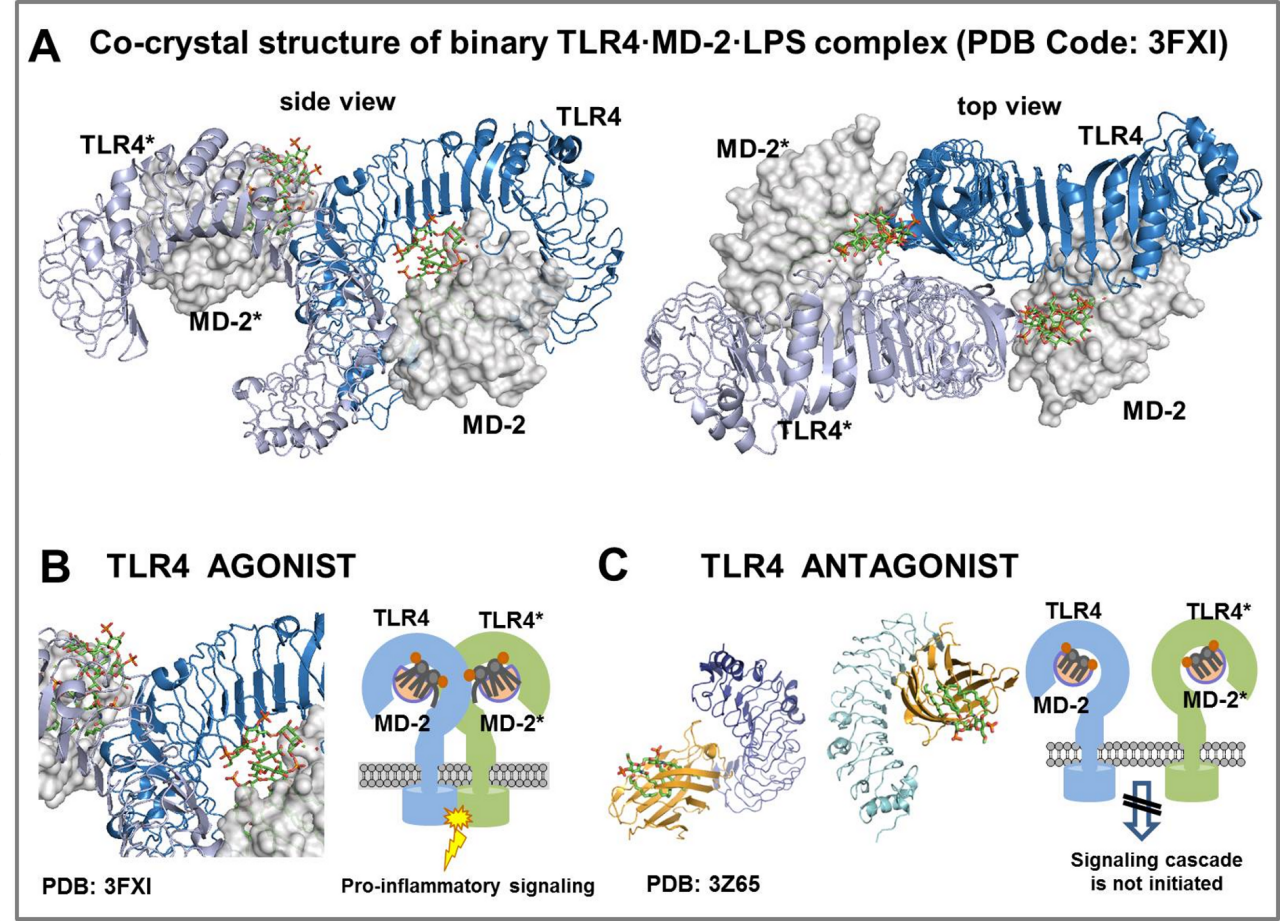
(A) Co-crystal structure of the homodimeric *E. coli* Ra-LPS·hMD-2∙TLR4 complex (PDB code: 3FXI); (B) schematic representation of the *E. coli* lipid A induced activation of the MD-2∙TLR4 complex (C) schematic representation of the interaction of TLR4 antagonist eritoran with MD-2∙TLR4 complex. Images were generated with PyMol, ChemDraw and PowerPoint.

Activation of the innate immune response by lipid A/LPS requires a consecutive interaction of lipid A with lipopolysaccharide-binding protein (LPB) [35], glycosylphosphatidylinositol-anchored surface protein CD14 (a differentiation antigen of monocytes) [36–37], followed by a soluble accessory protein MD-2 [38] and TLR4·MD-2 complex [39] (Figure 3) [40–44]. TLR4 is a germ-line encoded transmembrane protein composed of an ectodomain comprising leucin-rich-repeat motifs and a cytoplasmic domain responsible for the initiation of the pro-inflammatory signaling cascade. The lipid A portion of hexaacyl LPS (e.g., in *E. coli* LPS) is recognized and bound by a co-receptor protein MD-2 which is physically asssociated with TLR4. The binding of lipid A initiates dimerization of two copies of the TLR4∙MD-2–LPS complexes which results in the formation of a hexameric [TLR4∙MD-2–LPS]_2_ complex (Figure 3A). LPS-induced homodimerization of TLR4∙MD-2–LPS complexes provokes the recruitment of adaptor proteins to the cytoplasmic TIR (Toll/interleukin-1 receptor) domains of TLR4 which eventually results in the induction of the intracellular pro-inflammatory signaling and activation of the host innate immunity (Figure 3B) [42,45–46].

Compounds which compete with LPS in binding to the same site on MD-2 are capable of inhibiting the induction of the signal transduction pathway by preventing the LPS-induced receptor complex dimerization (Figure 3C). Application of natural or synthetic TLR4 antagonists represents one of the most effective approaches for down-regulation of the TLR4-mediated signaling. So far, several lipid A variants which can block the LPS-binding site on human (h)MD-2 have been identified: tetraacylated lipid IVa [47] and a non-pathogenic lipid A from *R. sphaeroides* [22,48], which served as structural basis for the synthetic antisepsis drug candidate eritoran [49–50]. Inadequate regulation of the TLR4-mediated signaling was recognized as crucial factor in the pathogenesis of chronic inflammatory, autoimmune and infectious diseases [51–53]. A number of studies also suggested a possible implication of TLR4 in cardiovascular disorders [54] and Alzheimer desease – associated pathology [55]. Therapeutic down-regulation of the TLR4 signaling is believed to be beneficial for treatment of numerous chronic and acute inflammatory diseases such as asthma [51], arthritis [52], influenza [50], and cancer [56]. Furthermore, TLR4 has been shown to link the innate and adaptive immunity [57–58], underscoring stimulation of the TLR4·MD-2 complex by non-toxic TLR4-specific ligands as an apparent tactic for development of novel vaccine adjuvants [59–61].

**Figure 4:**
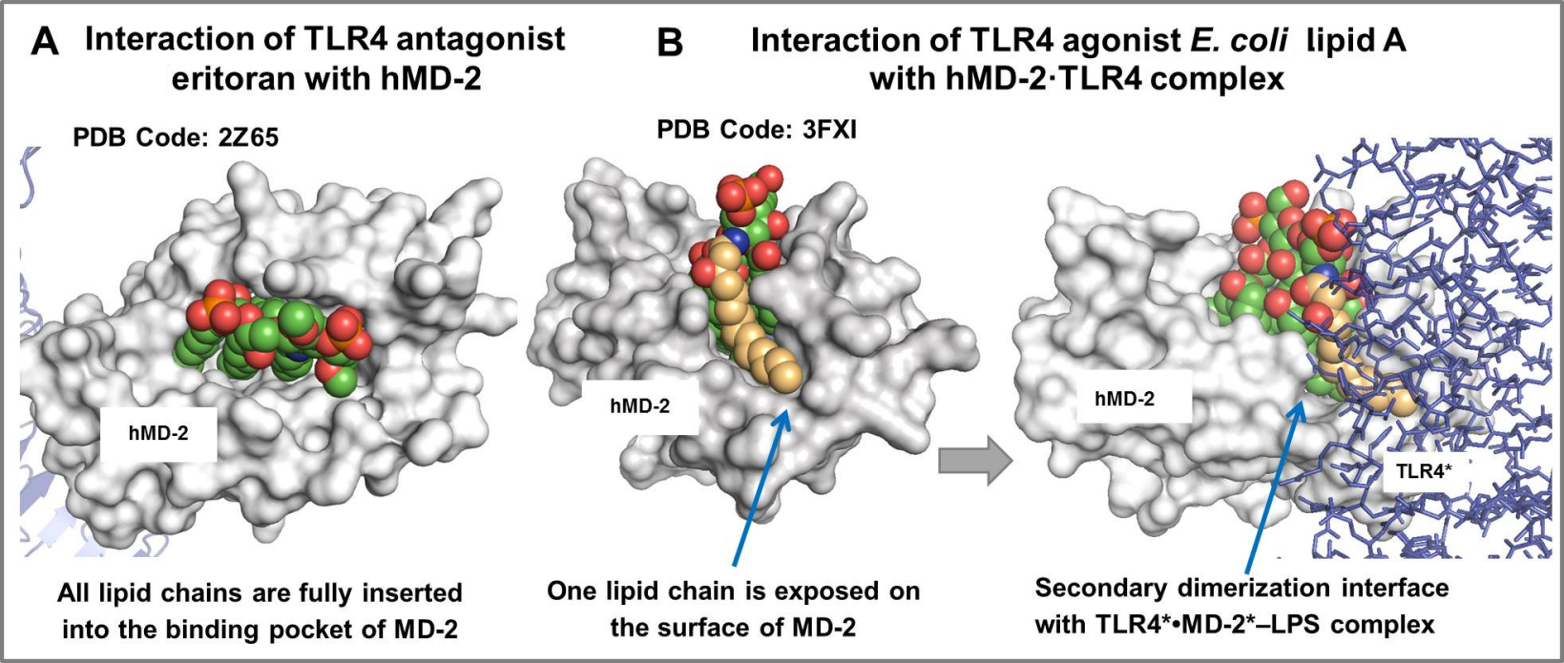
Co-crystal structures of (A) hybrid TLR4·hMD-2 with the bound antagonist eritoran (PDB: 2Z65, TLR4 is not shown); (B) homodimeric *E. coli Ra*-LPS·hMD-2∙TLR4 complex (PDB code: 3FXI, TLR4 is not shown, only lipid A portion is shown for clarity). Images were generated with PyMol.

X-ray structural analyses of the MD-2∙TLR4 complexes with bound variably acylated lipid A uncovered markedly different modes of interaction of agonist and antagonist TLR4 ligands. Commonly, the binding of hexaacylated bisphosphorylated lipid A (such as lipid A from *E. coli*) by the TLR4∙MD-2 complex results in an efficient activation of the innate immune response, while underacylated lipid A variants (such as tetraacylated lipid IVa [47], or a synthetic lipid A analogue eritoran) can block the endotoxic action of LPS [62–63]. All four acyl chains of antagonists eritoran and lipid IVa are fully inserted into the hydrophobic binding pocket of hMD-2 which results in an efficient binding without initiation of intracellular signaling (Figure 4A) [47,62]. In contrast, upon binding of hexaacylated *E. coli* LPS by the MD-2∙TLR4 complex, only five long-chain acyl residues of lipid A are interpolated into the binding pocket of MD-2, whereas the sixth 2*N*-acyl lipid chain is exposed onto the surface of the co-receptor protein, constituting the core hydrophobic interface (together with the Phe126 loop of MD-2) for the interaction with the second TLR4*∙MD-2*-LPS complex (Figure 4B) [42,64]. Thus, lipid A directly participates in the formation of an active multimeric ligand–receptor complex, whereas the tightness and efficiency of dimerization strongly depends on specific structural characteristics such as the acylation pattern and the number of negative charges (e.g., phosphate groups) in the molecule [65–67].

It has been just recently shown that TLR4 is not a sole receptor protein accountable for cellular responses induced by LPS. A number of pro-inflammatory effects such as autophagy, endocytosis and oxidative burst are induced by the LPS-mediated activation of an atypical inflammasome which is governed by the cytosolic enzyme caspase-11 and its human homologue caspase-4 [68]. Inflammasomes are protein complexes that are assembled in the cytosol of macrophages in response to the extracellular stimuli such as LPS [69]. The caspase-4/11 dependent inflammasomes are activated by the intracellular Gram-negative bacteria and largely contribute to development of endotoxic shock [70–71]. Biochemical studies revealed that caspase-4/11, which mediate inflammatory cell death by pyroptosis, are LPS receptors themselves [72–73].

Due to considerable micro-heterogeneity of the LPS isolates from wild-type or laboratory-adapted Gram-negative bacteria, the clinical and cellular studies as well as structure–activity relationship investigations using native LPS are complicated and difficult to evaluate. The lipid A content of LPS generally comprises a complex mixture of structural homologs having a variable number of the long-chain acyl residues of different chain lengths. The structural heterogeneity of lipid A preparations obtained through LPS isolation from bacterial cultures makes it difficult to get an unbiased correlation of specific structural features of lipid A and its TLR4-mediated activities. Moreover, possible contaminations with other pro-inflammatory bacterial components complicate the assessment of inflammatory pathways triggered by LPS in human and rodent immune cells. As example, not TLR4 but TLR2 (which mediates the host innate immune response to Gram-positive bacteria) was formerly reported to be responsible for the recognition of LPS belonging to certain bacterial strains. The micro-heterogeneity and contamination problem can be solved by application of synthetically prepared structurally defined lipid A variants of highest chemical and biological purity. To obtain clear structure–activity relationships data on lipid A–TLR4 interaction as well as unambiguous correlation of the lipid A acylation and phosphorylation pattern to its capacity in induction of different (i.e., MyD88-dependent and TRIF-dependent) signaling pathways, numerous well-defined lipid A substructures were synthesized. This review summarizes synthetic approaches developed in the past decade toward diverse LPS partial structures from different bacterial species including lipid A. The review provides comprehensive insight into the divergent and complex chemistry hidden under seemingly simple transformations needed for the assembly of lipid A, such as glycosylation towards fully orthogonally protected β(1→6)-linked diglucosamine backbone, sequential protective groups manipulation combined with successive instalment of multiple functional groups, *N-* and *O*-acylation with the long chain β-hydroxy fatty acids, anomeric phosphorylation and the synthesis of binary glycosyl phosphodiesters involving two amino sugars. Explicit structure–activity relationships data obtained with synthetic lipid A derivatives would also help to design novel therapeutic approaches for sepsis and inflammation.

## Review

### 1. Synthesis of *E. coli, N. meningitidis, S. typhimurium* and *H. pylori* LPS partial structures comprising lipid A

#### 1.1. Synthesis of *E. coli* and *S. typhimurium* lipid A

**Scheme 1:**
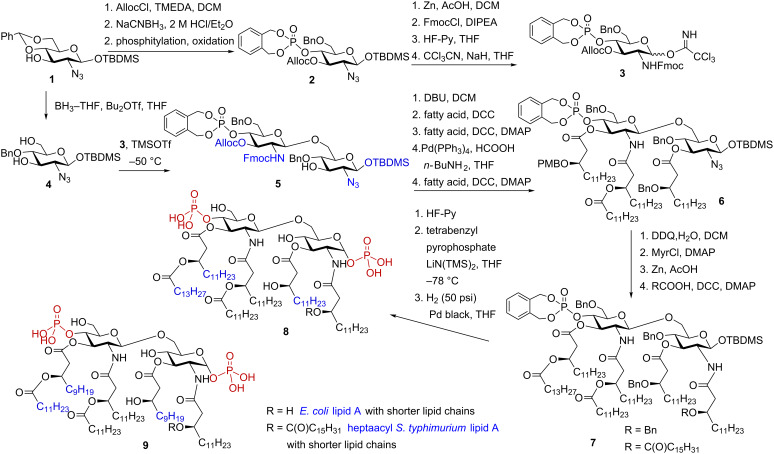
Synthesis of *E. coli* and *S. typhimurium* lipid A and analogues with shorter acyl chains.

*E. coli* and *S. typhimurium* lipid A’s count to the most powerful activators of the TLR4-mediated innate immune signaling and are responsible for the broad spectra of the inflammatory endotoxic effects in the infected host. To gain deeper insight into molecular basis of lipid A – TLR4 complex interaction and to determine the structural requirements for the efficient TLR4 activation, the hexaacylated lipid A corresponding to *E. coli* LPS, its analogue having 2 × CH_2_ shorter acyl chains at positions 3 and 3’ as well as heptaacylated *S. typhimurium* lipid A and the corresponding analogue with shorter lipid chains at C-3 and C-3’ were synthesised via a highly convergent synthetic route [74]. In contrast to previously developed approaches which employed donor and acceptor monosaccharide molecules that were already functionalized with the lipid chains and phosphate groups [75–76], the new synthetic route used orthogonally protected monosaccharide precursors **3** and **4** (Scheme 1).

The glycosyl donor **3** was synthesised starting from azide **1** by first protecting the 3-OH group with an allyloxycarbonyl (Alloc) protecting group followed by regioselective reductive opening of the 4,6-*O*-benzylidene acetal using NaCNBH_3_ and HCl in diethyl ether, and successive phosphitylation of the liberated 4’-OH functionality with *N,N*-diethylaminophosphepane (*N,N*-diethyl-1,5-dihydro-2,3,4-benzodioxaphosphepin-3-amine) in the presence of 1*H*-tetrazole followed by in situ oxidation with *m*-chloroperoxybenzoic acid (*m-*CPBA) to give fully protected 4’-phosphate **2**. The azido group in **2** was reduced, the resulting amine was converted to the *N*-Fmoc carbamate; the anomeric TBDMS ether was cleaved by treatment with HF in pyridine followed by reaction of the anomeric lactol with trichloroacetonitrile in the presence of a catalytic amount of NaH to provide trichloroacetimidate **3**. The glycosyl acceptor **4** was prepared from the same precursor **1** by regioselective reductive opening of benzylidene acetal using the borane−THF complex in the presence of Bu_2_BOTf. Regioselective TMSOTf-catalysed glycosylation of the diol **4** by the imidate donor **3** resulted in the formation of a single product, the β(1→6)-linked disaccharide **5**. After the 2’-*N*-Fmoc group in **5** was removed with DBU to provide a free amino group, the 2’-NH_2_ and 3-OH groups could be differentiated in the next acylation step by using DCC as activating agent for the *N*-acylation, and Steglich reaction conditions (DCC and DMAP) for the *O*-acylation. Following removal of the Alloc protecting group was readily performed by treatment with Pd(PPh_3_)_4_ in the presence of formic acid and butylamine to provide 3’-OH – containing precursor ready for the acylation by the long-chain acyloxyacyl acid. To avoid migration of the phosphotriester group from position 4’ to position 3’ and the formation of the acyloxy-chain elimination byproducts under DCC–DMAP-promoted acylation conditions, a two-step procedure for the acylation of 3’-OH group was applied. Acylation with the (*R*)-3-(*p*-methoxy)benzyloxytetradecanoic acid was initially performed to provide **6**, the (*p*-methoxy)benzyl ether was removed with DDQ and the liberated OH group was acylated with myristoyl chloride. Reduction of the 2-azido group by treatment with Zn in acetic acid followed by acylation of the amino group under standard conditions gave hexaacylated intermediate **7**.

The α-glycosyl phosphate was stereoselectively introduced by first, cleavage of the anomeric TBS ether by treatment with HF in pyridine, followed by phosphorylation using tetrabenzyl pyrophosphate in the presence of lithium bis(trimethyl)silylamide [76] in THF at −78 °C. Final deprotection by catalytic hydrogenolysis over Pd-black provided target lipid A derivatives **8** and **9** corresponding either to *E. coli* (R = H) and *S. typhimurium* (R = -C(O)C_15_H_31_) LPS with shorter acyl chains.

#### 1.2. Synthesis of *N. meningitidis* LPS partial structures including lipid A

There has been significant controversy in reports concerning the induction of the pro-inflammatory responses by *N. meningitidis* LPS and the differentiation of the intracellular TLR4-mediated signaling pathways (MyD88 vs TRIF) by its lipid A compared to *E. coli* lipid A. Indeed, differences in the acylation pattern (non-symmetric [4 + 2] for *E. coli* and symmetric [3 + 3] for *N. meningitidis)* and the length of acyloxyacyl lipid chains substituting positions 2’ and 3’ of the diglucosamine backbone (shorter for lipid A of *N. meningitidis*) could be responsible for such discrepancy. However, significant heterogeneity of biological preparations used for cellular in vitro experiments as well as the presence of possible biologically active contaminations in the isolated samples put the consistency of immunobiological evaluation at risk. Moreover, to decipher the mode of interaction of LPS with the TLR4 system, the analysis of cytokine induction profile generated by meningococcal Kdo- (3-deoxy-D-manno-oct-2-ulosonic acid) lipid A compared to synthetic unsubstituted *N. meningitidis* lipid A was essential. To achieve these aims, a facile synthesis of meningococcal lipid A and Kdo-lipid A was elaborated. By the time the synthesis was performed, the crystal structure of the homodimeric TLR4·MD-2·LPS complex was not yet solved and the information on the biological activity obtained with synthetic molecules was fundamental for the understanding the structural basis of endotoxin-protein interaction.

**Scheme 2:**
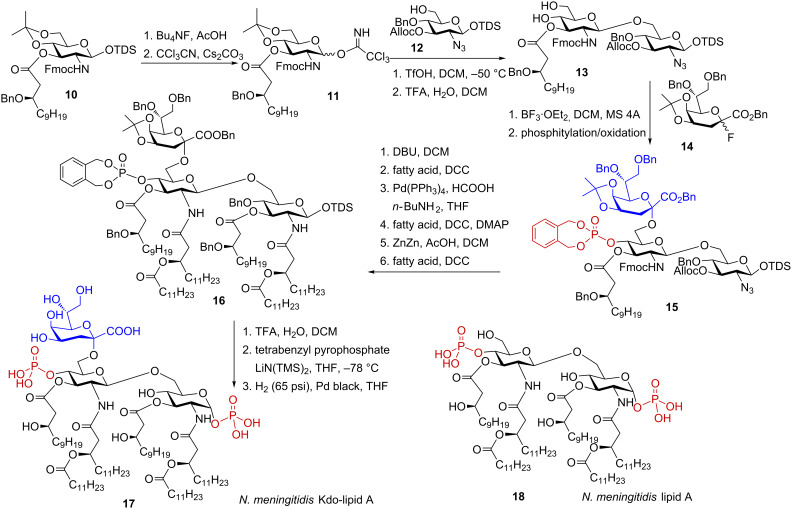
Synthesis of *N. meningitidis* Kdo-lipid A.

Preparation of Kdo-lipid A represents an even greater synthetic challenge than lipid A per se. The synthesis of *E. coli* type Kdo_2_-lipid A (*Re*-LPS) was performed earlier [77] and was previously reviewed [76]. The synthesis of *N. meningitidis* Kdo-lipid A entailed initial preparation of donor and acceptor molecules constituting the diglucosamine backbone [78]. Accordingly, the *N*-Fmoc protected thexyldimethylsilyl (TDS) derivative **10** was anomerically deprotected by treatment with tetrabutylammonium fluoride buffered with acetic acid, and the resulting lactol was converted to the imidate donor **11** which was coupled to the orthogonally protected acceptor, an azide **12**, using triflic acid as promotor (Scheme 2). Subsequent hydrolytic cleavage of the isopropylidene group furnished diol **13**. Regioselective boron trifluoride diethyl etherate-promoted glycosylation of the 6-OH group in **13** with Kdo-fluoride donor **14** afforded an inseparable mixture of α- and β-anomeric products (α/β = 9:1) [78]. Phosphitylation of the remaining OH group in position 4’ and facile separation of the anomeric α/β mixture furnished the anomerically pure trisaccharide **15**.

Next, three acyl residues were introduced at positions 2’, 3’ and 3 by successive deprotection–acylation sequence. The *N*-Fmoc protecting group was removed using DBU and the resulting free amino group was acylated with (*R*)-3-dodecanoyltetradecanoic acid in the presence of DCC as activating agent. Subsequently, the Alloc group was cleaved by treatment with Pd(PPh_3_)_4_ in the presence of BuNH_2_ and HCOOH and the resulting 3-OH group was acylated using DCC in the presence of DMAP as activating agent. Succeeding reduction of the azido function with zinc in acetic acid followed by acylation of the liberated amino group with the long-chain acyloxyacyl fatty acid furnished fully acylated **16**. In the next steps, the isopropylidene acetal and anomeric TDS ether were removed by treatment with aqueous TFA and the anomeric hydroxyl group was regio- and stereoselectively phosphorylated using tetrabenzyl diphosphate in the presence of lithium bis(trimethylsilyl)amide [76] to provide glycosyl phosphotriester as exclusively α-anomer. Global deprotection was accomplished by catalytic hydrogenolysis over Pd-black to give meningococcal Kdo-lipid A **17**. A lipid A derivative **18** lacking Kdo residue at position 6’ was prepared in a similar fashion.

Functional studies revealed that meningococcal Kdo-lipid A **17** was a much more potent inducer of the innate immune responses than lipid A **18** and stimulated the expression of TNF-α and IFN-β to a similar extent as its parent LPS. Thus, it could be confirmed, that lipid A having at least one Kdo residue attached at position 6’ of the diglucosamine backbone represents the minimum structural requirement needed for the full activation of the LPS-sensing receptor TLR4. Comparison of activities of synthetic meningococcal and enteric lipid A revealed that the former was more potent in the induction of expression of the pro-inflammatory cytokines which could be attributed to the differences in the acylation pattern in both molecules. Importantly, it was demonstrated that neither of synthetic lipid A derivative had a bias towards MyD88- or TRIF-dependent immune responses [78].

#### 1.3. Synthesis of fluorescent-labeled lipid A analogues

For studying the structural basis and the dynamics of TLR4-lipid A interplay, the application of labeled synthetic lipid A derivatives as versatile probes for tracking ligand–receptor interactions was exploited. However, the hydrophobic character and the large size of most fluorescent labels which could potentially compete with lipid A for the LPS binding site at the TLR4 complex, posed an additional challenge. The only optional hydroxyl group which could qualify as the site of attachment of a fluorescent label without hindering the biological activity would be position 6’ of the diglucosamine backbone of lipid A. When attached to position 6’ via a linker molecule, the fluorescent label would not interfere with the binding of lipid A to the MD-2·TLR4 complex, such that the full TLR4-mediated activity would be preserved. Accordingly, the 6’-*O*-glycine-linked BODIPY (4,4-difluoro-5,7-dimethyl-4-bora-3a,4a-diaza-s-indacene)-labeled lipid A was previously synthesized [79]. However, this compound revealed only a weak fluorescence in aqueous solution owing to enhanced amphiphilicity of the hybrid molecule inflicted by the hydrophobic character of the fluorescent label and the formation of aggregates which resulted in self-quenching.

**Scheme 3:**
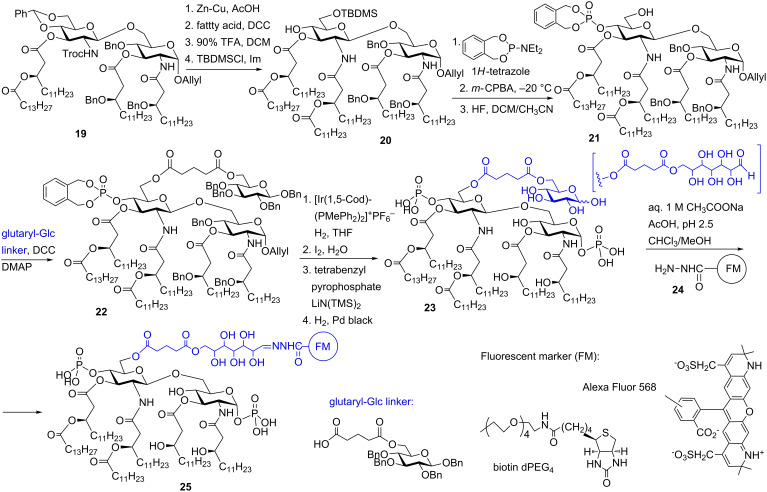
Synthesis of fluorescently labeled *E. coli* lipid A.

To circumvent these problems, a longer hydrophilic linker and a less hydrophobic fluorescent group were required. An elegant solution consisted in the application of glucose attached at position 6’ via a glutaryl group as a long-chain hydrophilic linker in combination with biotin or the hydrophilic fluorescent label AlexaFluor. The appropriately protected tetraacylated disaccharide **19** was subjected to treatment with Zn in AcOH which reductively cleaved the *N*-Troc group (Scheme 3). After *N*-acylation by (*R*)-3 acyloxyacyl fatty acid and hydrolytic cleavage of 4’,6’-*O*-benzylidene acetal group with 90% aqueous TFA, the liberated 6’-hydroxy group was regioselectively protected as TBDMS ether to furnish **20**. 1*H*-Tetrazole-catalysed phosphitylation of the 4’-OH group with *N,N*-diethylaminophosphepane followed by oxidation of the intermediate phosphite with *m-*CPBA to furnish the corresponding phosphate, and subsequent deprotection of the 6’-*O*-TBDMS ether gave the hexaacylated phosphotriester **21**.

The glutaryl-glucose linker (prepared from *O*-benzyl-protected glucose and glutaric anhydride) was introduced at the free 6’-OH group using DCC and DMAP to give **22**. The anomeric allyl group was cleaved by standard procedure, the phosphorylation of the 1-OH group was performed by 1-*O*-lithiation and subsequent treatment with tetrabenzyl pyrophosphate to furnish exclusively α-configured fully protected glycosyl phosphotriester. Global deprotection by catalytic hydrogenolysis over Pd-black gave *E. coli* lipid A functionalized with the glutaryl-Glc linker **23** which served as a key precursor for the preparation of fluorescent- or biotin-labeled compounds using labeling reagents having a hydrazide group.

A hydrophilic fluorescence group Alexa Fluor 568 and polyethylene glycol-linked biotin were introduced using hydrazone formation reaction between the aldehyde group of the glutaryl-Glc linker and the hydrazide group of the labeling reagent. In addition to labeled *E. coli* type lipid A **25**, the labeled tetraacylated lipid IVa was also prepared. Importantly, the bioactivity of labeled compounds was fully preserved (the labeled *E. coli* type lipid A **25** performed as strong TLR4 agonist and the labeled tetraacylated lipid IVa acted, as expected, as TLR4 antagonist) and the fluorescence intensity of **25** and its tetraacylated counterpart was comparable with the fluorescence of the labeling reagent alone. Aggregation-mediated fluorescence quenching was not observed which confirmed the advantage of application of highly hydrophilic linker molecules and non-hydrophobic labeling reagents for amphiphilic glycoconjugates such as lipid A.

#### 1.4. Synthesis of *Helicobacter pylori* Kdo-lipid A substructures

A *Helicobacter pylori* infection of the gastric mucosa causes chronic gastritis in humans and plays a pivotal role in the progression and pathogenesis of peptic ulcer diseases. Persistent infection with *H. pylori* is implicated in the development of gastric carcinoma [80]. *H. pylori* colonizes about 50% of the world’s population and can asymptomatically persist for decades within a single host. The infection with *H. pylori* inevitably results in a chronic inflammatory response, whereas *H. pylori* LPS-dependent activation of monocytes and gastric epithelial cells leads to the production of several pro-inflammatory cytokines and reactive oxygen species (ROS) [81]. The mechanism by which *H. pylori* induces chronic inflammation and injury of gastric tissue is not fully understood. *H. pylori* produces a unique LPS molecule notable for strikingly low endotoxicity which is attributed to the structure of its lipid A moiety [81]. *H. pylori* uses two constitutive lipid A-mediated evasion strategies: repulsion of CAMPs (which are present at high concentrations in the gastric mucosa) and evasion of detection by the TLR4 system. Similarly to enteric *E. coli* LPS, *H. pylori* produces hexa-acylated lipid A, however, it displays a tetra- and triacylated lipid A molecule lacking the 4’-phosphate group on the bacterial surface [82–83]. Reduced number of acyl chains and the absence of the phosphate group at position 4’ prevent detection of LPS by the TLR4. Thus, owing to posttranslational modifications performed by several enzymes, the lipid A of *H. pylori* is poorly recognized by the innate immune system of the host [84]. The 1-phopshate group of *H. pylori* lipid A is further masked with ethanolamine that reduces the net negative charge and induces resistance to CAMPs (Figure 2). The unique structure of *H. pylori* lipid A plays a pivotal role in evading the host immune response by the bacterium [84]. Synthetically prepared structurally defined homogeneous *H. pylori* lipid A should help to identify the factors responsible for chronic inflammation during *H. pylori* infection.

**Scheme 4:**
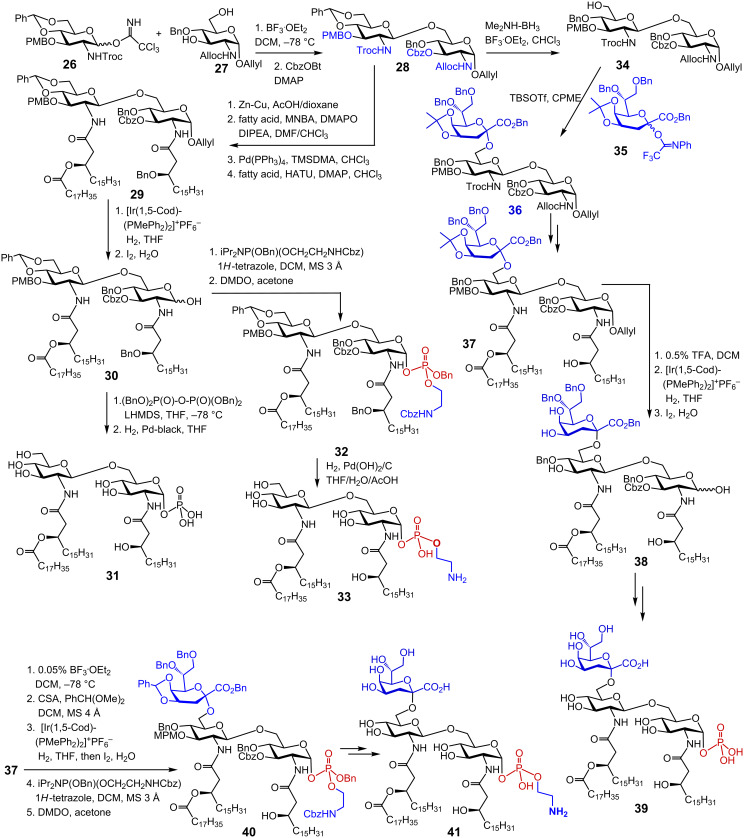
Synthesis of *H. pylori* lipid A and Kdo-lipid A.

The syntheses of *H. pylori* lipid A structures wherein the anomeric position was not modified with phosphoethanolamine were previously undertaken [85–86]. The syntheses of more sophisticated *H. pylori* lipid A substructures substituted by one Kdo residue at position 6’ and/or modified with ethanolamine at the glycosidic phosphate were accomplished just recently [21,87–88]. The synthetic strategy relied on the initial preparation of fully orthogonally protected βGlcN(1→6)GlcN disaccharide which was then stepwise functionalized with a variable number of the long-chain (*R*)-3-acyloxy- and (*R*)-3-acyloxyacyl residues, 1-*O*-phosphate or 1-*O*-phosphoethanolamine groups and a 6’-linked Kdo moiety [21,88]. The synthesis commenced with the preparation of donor **26** and acceptor **27** molecules, which were coupled using BF_3_·OEt_2_ as promotor to furnish fully protected β(1→6) diglucosamine (Scheme 4). Subsequently, the 3-OH functionality was protected with a carboxybenzyl group to give the key disaccharides **28**. The *N*-Troc group was reductively cleaved with Zn/Cu in acetic acid followed by acylation of the liberated 2’-amino group with the corresponding fatty acid using 2-methyl-6-nitrobenzoic anhydride (MNBA) as activating reagent in the presence of the nucleophilic catalysts 4-(dimethylamino)pyridine *N*-oxide (DMAPO) [89]. Next, the 2-*N*-Alloc group was cleaved by treatment with Pd(PPh_3_)_4_ and dimethylaminotrimethylsilane (TMSDMA) [90], followed by protection of the liberated 2-amino group by reaction with (*R*)-3-benzyloxycarboxylic acid using *O*-(7-azabenzotriazol-1-yl)-*N,N,N′,N′-*tetramethyluronium hexafluorophosphate (HATU) and DMAP as coupling reagents which furnished triacylated precursor **29**.

The 1-*O*-allyl group was then isomerized in the presence of an Ir complex and the resulting prop-1-enyl group was then removed by aqueous iodine to yield hemiacetal **30** which was stereoselectively phosphorylated by reaction with lithium hexamethyldisilazide (LHMDS), and subsequent treatment with tetrabenzyl pyrophosphate. Final deprotection by catalytic hydrogenation furnished lipid A **31**. Alternatively, the lactol **30** was phosphitylated by application of the phosphoramidite procedure with (benzyloxy)[(*N-*Cbz-3-aminopropyl)oxy](*N,N*-diisopropylamino)phosphine in the presence of 1*H*-tetrazole and subsequent oxidation with dimethyldioxirane (DMDO) [91] to furnish protected lipid A derivative **32**. Global deprotection by hydrogenation over Pd(OH)_2_/C in the presence of acetic acid afforded ethanolamine-modified *H. pylori* lipid A **33**.

To get deeper insight into the immunomodulatory potential of *H. pylori* lipid A, an access to synthetic *H. pylori* Kdo-lipid A was necessary. The presence of the Kdo moiety was shown to be decisive for the expression of full TLR4-mediated activity of lipid A. Previously, an efficient glycosylation strategy toward *E. coli* Kdo-lipid A using Kdo fluorides was developed by the same group. Glycosylation with Kdo fluoride required an excess of Lewis acid as promotor which was incompatible with the acid-labile protecting groups present in the key diglucosamine precursor. Therefore, a new *N*-phenyltrifluoroacetimidate Kdo donor **35** was developed (Scheme 4) [21]. The disaccharide acceptor **34** was prepared by regioselective reductive opening of 4′,6′-*O*-benzylidene acetal in **28** with Me_2_NH·BH_3_ and BF_3_·OEt_2_ in chloroform as solvent. The glycosylation of **34** with Kdo donor **35** was performed in CPME ether in the presence of TBSOTf as promotor to result in the stereoselective formation of trisaccharide **36**. Alternative microfluidic conditions applied by the authors ensured even better stereoselectivity and higher yields [21]. Sequential protective group manipulation and *N*-acylation procedure furnished the lipid A precursor **37**. The isopropylidene and anomeric allyl groups in **37** were removed and the anomeric position in **38** was regioselectively phosphorylated in a stereoselective manner by 1-*O*-lithiation with LHMDS, and subsequent treatment with tetrabenzyl pyrophosphate at −78 °C. Protecting groups were removed by hydrogenolysis on Pd-black to give *H. pylori* lipid A **39**. For the synthesis of Kdo-lipid A **41** entailing a phosphoethanolamine group at the anomeric position, the isopropylidene group in **37** had to be exchanged for the benzylidene group to avoid an application of acidic hydrolysis conditions for final deprotection of the labile glycosyl phosphodiester. After removal of the 1-*O-*allyl group using standard conditions, the anomeric lactol was phosphorylated via phosphoramidite procedure to furnish fully protected trisaccharide phosphodiester **40**, which was deprotected by hydrogenolysis on Pd(OH)_2_/C in THF/H_2_O/AcOH to give *H. pylori* lipid A **41**.

The availability of pure homogeneous synthetic compounds allowed for extensive immunobiological studies which revealed the unique functional properties of *H. pylori* lipid A. Triacylated lipid A variants efficiently inhibited the expression of IL-1β, IL-6 and IL-8 induced by *E. coli* LPS in human peripheral whole blood cells and the Kdo-containing lipid A substructures revealed the highest antagonist activity. On the other hand, all synthetic *H. pylori* lipid A and Kdo-lipid A showed IL-18 and IL-12 inducing activity, whereas the presence of Kdo decreased the potencies. Thus, it was shown that underacylated *H. pylori* lipid A could disrupt the TLR4-mediated NF-κB signaling by inhibiting the LPS-triggered release of IL-6 and IL-8 and, at the same time, could activate other signaling pathways resulting in the induction of IL-12 and IL-18. This unique immunomodulating feature of *H. pylori* lipid A was linked to bacterial ability to dampen the acute immune reaction of the host and promote chronic inflammation.

### 2. Synthesis of lipid A containing unusual lipid chains or lacking 1-phosphate group

#### 2.1. Synthesis of variably acylated *Porphyromonas gingivalis* lipid A

*Porphyromonas gingivalis* is a major bacterial pathogen strongly implicated in periodontal disease (periodontitis) that is the primary cause of tooth loss in adults worldwide. Increasing evidence suggest that *P. gingivalis* contributes to augmented systemic level of inflammation by invading the gingiva and modulating the innate inflammatory responses of the host which links periodontitis to various systemic diseases such as diabetes and cardiovascular disorders. The LPS of *P. gingivalis*, and particularly its lipid A, is recognized as major PAMP implicated in the pathogenesis of the periodontal disease. *P. gingivalis* LPS has been shown to stimulate the persistent production of IL-1, IL-6, and IL-8 in gingival fibroblasts which are thought to contribute to tissue destruction in gingivitis. On the other hand, it was demonstrated that *P. gingivalis* abolishes the expression of IL-8 in gingival epithelial cells which obstructs the host's capacity to recruit neutrophils to the sites of infection. Moreover, monocytes and human endothelial cells exhibit a low responsiveness to *P. gingivalis* LPS compared to *E. coli* LPS. *P. gingivalis* LPS was even shown to directly compete with *E. coli* LPS at the TLR4 complex in human endothelial cells, thus acting as TLR4-dependent antagonist of *E. coli* LPS. These discrepancies could be explained by a significant amount of structural heterogeneity displayed by *P. gingivalis* LPS containing both three-, tetra- and pentaacylated lipid A species [92]. The effects of these isoforms of *P. gingivalis* LPS on the expression of IL-6, IL-8 and TNF-α in human gingival fibroblasts are vastly diverse which contributes to periodontal pathogenesis [93–94]. Another structural peculiarity of the lipid A of *P. gingivalis* consists in the presence of the unusual branched fatty acid residues: *R*-(3)-hydroxy-13-methyltetradecanoate and *R*-(3)-hydroxy-15-methylhexadecanoate, which are non-symmetrically distributed across the diglucosamine backbone. Strong controversies in assessment of biological activities of *P. gingivalis* lipid A based on the LPS isolates [95–97] prompted chemical synthesis of structurally defined variably acylated *P. gingivalis* lipid A substructures [98–99].

**Scheme 5:**
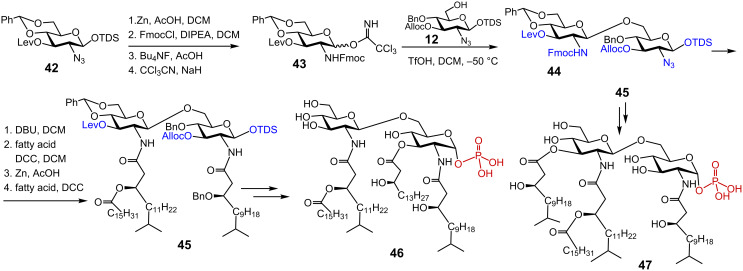
Synthesis of tetraacylated lipid A corresponding to *P. gingivalis* LPS.

Tetraacylated lipid A substructures representing the major lipid A of *P. gingivalis* were synthesised through a highly convergent approach employing a fully orthogonally protected key disaccharide **44** [98] (Scheme 5). A combination of temporary 3’-*O*-levulinoyl (Lev), 3-*O*-allyloxycarbonyl (Alloc) and 1-*O*-hexyldimethylsilyl (TDS) protecting groups with permanent benzyl/benzylidene acetal protections for hydroxyl groups and application of 9-fluorenylmethoxycarbamate (Fmoc) and azido protecting groups for masking the NH_2_ functionalities allowed for the stepwise instalment of functional groups (phosphates and fatty acids) into the diglucosamine **44**. For the assembly of key disaccharide **44**, the azido group in **42** was exchanged for the *N*-Fmoc group by reduction with Zn in AcOH and reaction with FmocCl; anomeric TDS ether was cleaved and the resulting lactol was converted into the imidate donor **43** (Scheme 5). Glycosylation of the free 6-OH group in the acceptor azide **12** with the imidate donor **43** furnished fully orthogonally protected βGlcN(1→6)GlcN **44**. Next, the 2’-*N*-Fmoc group in **44** was removed by treatment with DBU and the first unusual branched acyloxyacyl residue was installed. For the preparation of (*R)*-3-hydroxy-13-methyltetradecanoic and (*R*)-3-hexadecanoyloxy-15-methylhexadecanoic acids an efficient cross-metathesis has been employed [98]. Reduction of the 2-azido group with Zn in acetic acid, followed by acylation with the respective 3-*O*-benzyl protected fatty acid provided the key intermediate **45**. Sequential protecting group manipulation (3’-*O*-Lev, 3-*O*-Alloc and 1-*O*-TDS) combined with acylation and regioselective anomeric phosphorylation furnished, after global deprotection, variably acylated *P. gingivalis* lipid A substructures **46** and **47**. The synthetic compounds did not stimulate the NF-κB signaling pathway, but efficiently inhibited the LPS-induced production of TNF-α in human monocytes. The acylation pattern was found to be decisive for the expression of the antagonist activity since 2’,3,2-triacylated lipid A **46** was a more potent antagonist than its 2’,3’,2-triacylated counterpart **47**.

**Scheme 6:**
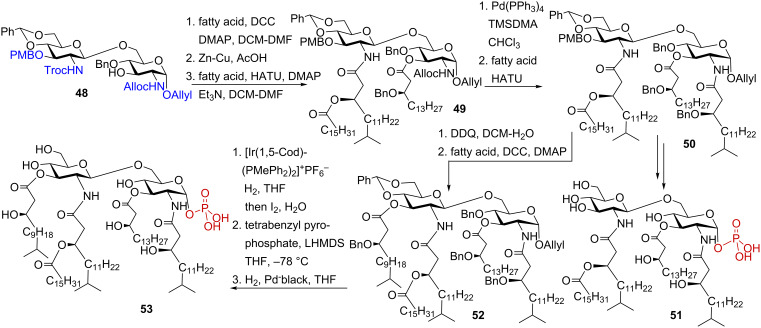
Synthesis of pentaacylated *P. gingivalis* lipid A.

Synthesis of the *P. gingivalis* pentaacyl lipid A was based on the initial preparation of the orthogonally protected glucosamine disaccharide **48** [99]. Initial acylation of the free OH group in position 3, followed by sequential manipulation of the amino-protecting groups (2’-*N*-Troc and 2-*N*-Alloc) and acylation with the corresponding branched (*R*)-3-benzyloxyacyl and (*R*)-3-acyloxyacyl fatty acids furnished the lipid A precursor **50** (Scheme 6). Cleavage of the 1-*O*-allyl protecting group and stereoselective phosphorylation of the anomeric position via 1-*O*-lithiation with LHMDS, and subsequent treatment with tetrabenzyl pyrophosphate gave tetraacylated *P. gingivalis* lipid A **51**. For the synthesis of pentaacyl lipid A **53**, the 3’-*O*-*p*-methoxybenzyl group in **50** was cleaved by treatment with DDQ, and the liberated hydroxyl group was reacted with branched β-benzyloxy fatty acid to furnish fully acylated precursor **52**. After the cleavage of the 1-*O*-allyl group, the resulting lactol was phosphorylated to provide exclusively α-configured anomeric phosphotriester, which, after final deprotection by hydrogenolysis, gave pentaacyl lipid A **53**.

Immunobiological studies revealed that synthetic tri- and tetraacylated *P. gingivalis* lipid A substructures efficiently inhibited cytokine production induced by *E. coli* LPS, whereas the pentaacylated compound was less efficient in antagonizing LPS-mediated inflammatory responses. Interestingly, tetraacylated **51** selectively induced the expression of IL-18 which could be characteristic for LPS from bacteria causing asymptomatic chronic infection and persistent inflammation.

#### 2.2. Synthesis of monophosphoryl lipid A (MPLA) as potential vaccine adjuvant

In contrast to the attenuated or whole killed vaccines which contain bacterial cell wall components and nucleic acids serving as naturally occurring adjuvants, the subunit vaccines lack these components. In the last decade much attention has been focused on the development of adjuvants that can render subunit vaccines more efficient by boosting the adaptive immune response. In this respect, TLR agonists deserved special consideration, since the induction of the innate immune signaling with PAMPs was shown to greatly enhance the adaptive immune responses [100].

Monophosphoryl lipid A (MPLA), an efficient and safe vaccine adjuvant registered for the use in Europe [59] is derived from the LPS of *Salmonella minnesota* R595 by following chemical modifications: elimination of the core oligosaccharide, hydrolysis of the 1-phosphate from the reducing end glucosamine, and removal of the acyl chain from position 3 of the disaccharide backbone [59]. Lower toxicity of the TLR4 ligand MPLA compared to its parent LPS/lipid A was linked to the absence of the phosphate group in position 1 of the diglucosamine backbone [101–102]. The absence of the 1-phosphate group on the MPLA molecule weakens the efficiency of the homodimerization of two TLR4·MD-2-ligand complexes which results in a weaker cytokine inducing capacity, diminished immune activation and lower endotoxic activity, while retaining immunogenicity [103]. MPLA differs from *E. coli* lipid A not only by the absence of the 1-phosphate group, but also in the acylation pattern. MPLA was reported to induce the innate immune response via a TRIF-mediated signaling pathway (in contrast to enteric lipid A which activates MyD88 pathway) [104]. A recent study demonstrated that both TLR4 and MyD88 signaling have a significant effect on the adaptive immune responses in MPLA-adjuvanted vaccines [105]. To gain deeper understanding of the mechanisms underlying beneficial non-toxic immune response induced by MPLA and to reveal the major structural requirements responsible for adjuvant activity, monophosphoryl lipid A and several analogues differing in the acylation pattern have been synthetically prepared [106–107].

**Scheme 7:**
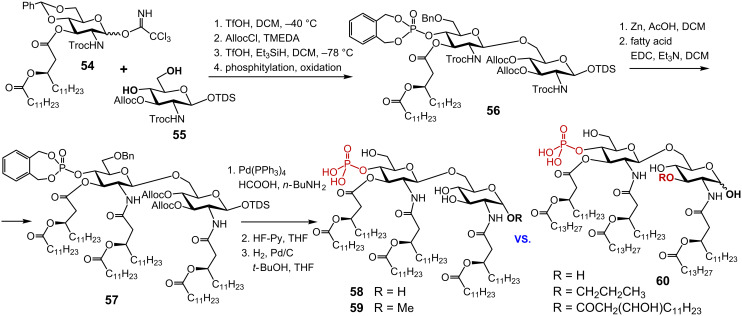
Synthesis of monophosphoryl lipid A (MPLA) and analogues.

The synthesis of MPLA equipped with shorter secondary acyl chains **58** was achieved via regioselective glycosylation of the primary hydroxy group at position 6 in the *N*-Troc-protected acceptor **55** by the imidate donor **54** (Scheme 7) [106]. The (*R*)-3-dodecanoyloxytetradecanoyl residue was preinstalled in position 3 of the GlcN donor molecule. Acylation by an acyloxyacyl fatty acid at the latter stage of the synthesis could result in phosphate migration and/or elimination of the secondary acyl chain. TfOH-mediated 1,2-*trans* glycosylation smoothly provided β(1→6)-linked diglucosamine, the free OH group in position 3 was protected as Alloc carbonate and the benzylidene acetal protecting group was regioselectively reductively opened to furnish 6’-*O*-benzyl ether. The liberated 4’-OH group was phosphorylated via phosphoramidite procedure to furnish **56**. Next, both 2- and 2’-*N*-Troc groups were reductively cleaved using Zn in acetic acid and the resulting 2’- and 2-amino groups were acylated with (*R*)-3-dodecanoyltetradecanoic acid to give **57**. Three types of protecting groups – allyloxycarbonyl (Alloc), hexyldimethylsilyl (TDS) and benzyl – were sequentially removed to provide the target compound **58**. A monophosphoryl lipid A analogue **59** wherein the anomeric center of the proximal GlcN moiety is modified as methyl glycoside was prepared in a similar fashion.

It was expected that the small methyl group substituting the anomeric OH functionality would not compromise biological activity. Both MPLA analogues **58** and **59** were less efficient in eliciting TNF-α in mouse macrophages compared to a commercially available *S. minnesota* MPLA preparation, whereas methyl glycoside **59** showed somewhat higher pro-inflammatory activity. Interestingly, attachment of varying 3-*O*-substitutions at position 3 of the reducing GlcN moiety in MPLA analogue **60** did not enhance the adjuvant activity [107].

Importantly, synthetic MPLA derivatives having variable acylation pattern were successfully utilized as build-in-adjuvants in fully synthetic self-adjuvanting glycoconjugate cancer vaccines [108–110].

#### 2.3. Synthesis of lipid A from *Rhizobium sin-1*

The *Rhizobiaceae* family refers collectively to the group of Gram-negative nitrogen-fixing plant endosymbiont bacteria. Lipid A of *Rhizobium* displays several significant structural differences when compared with *E. coli* lipid A: it lacks phosphate groups, but contains a galacturonic acid residue at the 4′-position and an aminogluconate moiety in place of the usual glucosamine 1-phosphate unit [111]. *Rhizobium* lipid A is esterified with a peculiar long chain fatty acid, 27-hydroxyoctacosanoate, which is not found in enteric Gram-negative bacteria [112]. The biosynthesis of lipid A in *R. leguminosarum* proceeds under the action of the same enzymes as in *E. coli* to generate the conserved phosphate containing precursor, Kdo_2_-lipid IVa. Several additional enzymes, namely 1-phosphatase and 1-oxidase, catalyze further conversion of Kdo_2_-lipid IVa into *R. leguminosarum* lipid A. The 1-phosphatase cleaves the 1-phosphate group to generate glucosamine which is subsequently converted to 2-amino-2-deoxygluconate in an oxygen dependent manner via the action of an oxidase located in the outer membrane [113–114].

The unique *Rhizobium* lipid A lacks the structural features which are necessary for the TLR4-mediated stimulation of the innate immune system in animals. This might conceivably help bacteroids to evade the innate immune response in plants during symbiosis in root cells. Additionally, certain *Rhizobium sin-1* lipid A isolates were shown to inhibit the LPS induced toxic effects in human immune cells [115]. To determine the structural features which are responsible for the LPS antagonizing properties of the heterogeneous *Rhizobium sin-1* lipid A preparations, the synthesis of several defined *Rhizobium* lipid A structures has been undertaken [116–119].

**Scheme 8:**
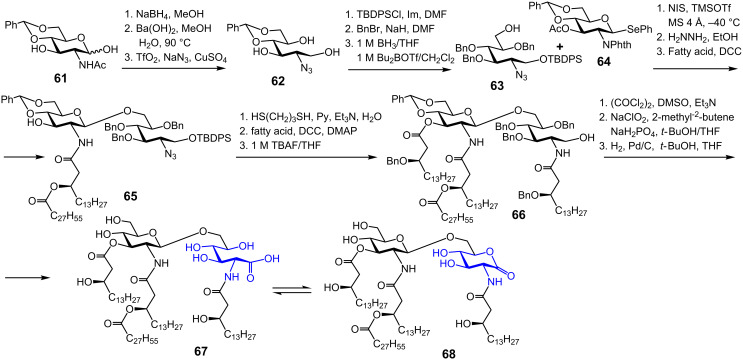
Synthesis of tetraacylated *Rhizobium* lipid A containing aminogluconate moiety.

A convergent synthetic approach towards *Rhizobium* lipid A substructures, 2-aminogluconate **67** and 2-aminogluconolactone **68**, included initial preparation of the alditol **62** (Scheme 8) [118]. To this end, GlcN hemiacetal **61** was reduced by treatment with NaBH_4_, the acetamido group was removed with barium hydroxide, and the resulting amine was transformed into azide **62**. The primary alcohol in **62** was regioselectively protected as silyl ether, followed by benzylation and reductive opening of the benzylidene acetal to give the acceptor monosaccharide **63**. NIS/TMSOTf-promoted glycosylation of **63** with glycosyl donor **64** furnished desired β(1→6) disaccharide which was subjected to treatment with hydrazine hydrate to remove the phthalimido group. Subsequent acylation of the liberated NH_2_ group provided **65**. A successive protective group manipulation/acylation sequence furnished tetraacylated **66**.

The oxidation of the primary alcohol in **66** to form the corresponding carboxylic acid was achieved by a two-step procedure involving oxidation under Swern conditions to give an intermediate aldehyde that was immediately subjected to a second oxidation with NaClO_2_ and sodium dihydrogen phosphate to afford the 2-aminogluconate. In a final step, the benzyl ethers and the benzylidene acetal protecting group were removed by hydrogenolysis over Pd/C to give **67**. After the 2-aminogluconolactone **68** was separately synthesized, the NMR spectra of **67** and **68** were found to be identical indicating the co-existence of both forms in neutral conditions. Thus, it was demonstrated that *Rhizobium* lipid A exists in an equilibrium between open- and closed-ring forms, namely, as a mixture of 2-aminogluconate **67** and 2-aminogluconolactone **68**.

**Scheme 9:**
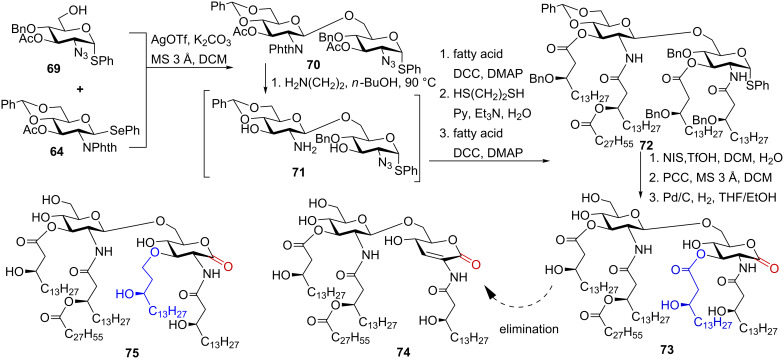
Synthesis of pentaacylated *Rhizobium* lipid A and its analogue containing ether chain.

In an effort to develop more potent TLR4 antagonists, the synthesis of pentaacylated *R. sin-1* lipid A as well as its analogue modified by an ether-linked lipid chain in position 3 was undertaken [116–117]. High-yielding chemoselective coupling of the thioglycoside acceptor **69** with selenoglycoside donor **64** gave the disaccharide **70** (Scheme 9). Sequential removal of the amino-protecting groups (phthalimido group with ethylenediamine in refluxing butanol to furnish **71**, and the azido group by reduction with propane-1,3-dithiol) and subsequent acylation with respective fatty acids provided pentaacyl compound **72**. Hydrolysis of the thiophenyl moiety was performed by treatment with *N*-iodosuccinimide (NIS) and a catalytic amount of trifluoromethanesulfonic acid in wet dichloromethane, the benzyl ethers and benzylidene acetal were removed by catalytic hydrogenation on Pd/C to give *Rhizobium* lipid A **73**.

Biological evaluation of the synthetic *R. sin-1* lipid A **73** was complicated by its chemical lability owing to extensive elimination which gave the enone derivative **74**. To circumvent this problem, the β-hydroxy ester at C-3 of the proximal GlcN unit in **73** was replaced by an ether lipid chain to furnish *R. sin-1* lipid A analogue **75** [117].

Cellular activation studies revealed that synthetic *R. sin-1* lipid A was 100-fold less potent than its parent LPS in inducing TNF-α and IFN-β in murine macrophages. Interestingly, the difference in the TLR4 activation potencies between LPS and lipid A was much more pronounced for *E. coli* LPS (LPS was 10000-fold more active than the corresponding lipid A) than for *R. sin-1* LPS and lipid A (100-fold). No cytokine release was measured for 3-ether analogue **75**, however, **75** was nearly as active as **73** in inhibiting TNF-α and IP-10 production induced by *E. coli* LPS in human monocytes [117]. Thus, *R-sin 1* lipid A **73** and **75** antagonized the expression of cytokines resulting from both MyD88- and TRIF-dependent signaling pathways in human monocytic cell line indicating that the exchange of 3-ester linkage for the 3-ether linkage has only marginal impact on the TLR4 antagonizing activity. However, this difference exerted a dramatic effect on the species specific activation of cellular responses in murine macrophages wherein compound **73** induced the release of pro-inflammatory cytokines and the *R-sin 1* lipid A analogue **75** was inactive.

**Scheme 10:**
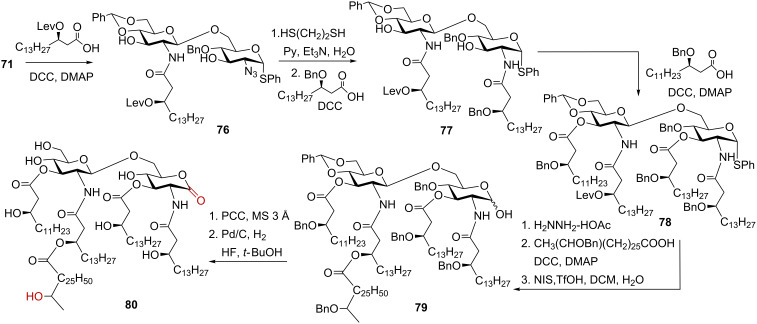
Synthesis of pentaacylated *Rhizobium* lipid A containing 27-hydroxyoctacosanoate lipid chain.

To determine the impact of hydroxylation of the long-chain 27-hydroxyoctacosanoic acid moiety for antagonist properties of *R-sin 1* lipid A, a lipid A containing this unique acyl residue was synthesised (Scheme 10). 27-Hydroxyoctacosanoic acid was prepared by employing a cross-metathesis between the ω-unsaturated ester and 3-butene-2-ol in the presence of Grubbs’ second generation catalyst [119]. An appropriately protected disaccharide **71** having free amino group in position 2’ was acylated by 3-*O*-levulinoyl protected (*R*)-3-hydroxyhexadecanoic acid [120] which, after the cleavage of levulinoyl protecting group, was esterified with benzyl ether protected 27-hydroxyoctacosanoic acid. Such a two-step approach facilitated the installment of the 27-hydroxyoctacosanoic residue into the lipid A moiety, and allowed for the synthesis of a series of differently acylated lipid A derivatives [119]. The azido group in monoacylated **76** was reduced with 1,3-propane dithiol, and the resulting amine was regioselectively acylated to give **77**. The free 3- and 3’-OH groups were acylated with (*R*)-3-benzyloxytetradecanoic acid under Steglich conditions to provide **78**, followed by cleavage of the levulinoyl ester and installment of the secondary ω-hydroxy acyl chain to furnish, after deprotection of the anomeric center, the hemiacetal **79**. The mixture of anomeric lactols was oxidized with pyridinium chlorochromate (PCC) to furnish the corresponding lactone, followed by hydrogenolysis on Pd/C to provide the target *R-sin 1* lipid A **80**.

### 3. Synthesis of aminosugar modified lipid A: the assembly of binary glycosyl phosphodiesters

#### 3.1. Synthetic challenges in the assembly of 1,1’-glycosyl phosphodiesters

Most naturally occurring glycosyl phosphodiesters entail the phosphoester linkage connecting one anomeric and one solely non-anomeric hydroxyl group. The assembly of such phosphodiesters is universally carried out using P(V)-based phosphotriester method, or P(III)-based phosphoramidite or H-phosphonate approaches [121–123]. In rare cases, however, the phosphodiester linkage can link the anomeric centers of two aminosugars as in the lipid A moieties of *Burkholderia, Bordetella* and *Francisella* LPS. The stereoselective assembly of 1,1′-glycosyl phosphodiesters represents a demanding synthetic challenge with respect to the necessity for the double anomeric stereocontrol and the inherent lability of the glycosyl phosphate intermediates. Generally, two major approaches can be applied for the synthesis of double glycosyl phosphodiesters, specifically, the phosphoramidite and the H-phosphonate procedures which are notorious for the mildness of the reaction conditions and the high reactivity of the P(III)-based intermediates. A three-coordinated phosphoramidite or a tetra-coordinated H-phosphonate species possess an electrophilic phosphorus centre which can instantly react with various nucleophiles. The benefits of the phosphoramidite methodology involve the mildness of the phosphitylation and oxidation conditions, while the chemical instability of the intermediary glycosyl phosphoramidites and glycosyl phosphites belongs to the drawbacks. For instance, isolation of the extraordinary labile glycosyl phosphoramidite intermediates in anomerically pure form looks rather unfeasible. The benefits of the H-phosphonate procedure rely on the stability of the glycosyl H-phosphonate monoesters which can be readily isolated by silica gel column chromatography, as well as on the absence of a protecting group at the phosphorus atom. Yet, the classic pivaloyl chloride (PivCl)-mediated H-phosphonate coupling reaction can result in the formation of a number of byproducts, and in the hydrolysis of the target 1,1´-glycosyl phosphodiester upon harsh conditions of aqueous iodine-mediated oxidation of the intermediate P(III) H-phosphonate phosphodiesters into the P(V) species. Fortunately, expedient modification of the H-phosphonate technique in terms of application of alternative coupling and oxidative reagents renders it to the method of choice for the assembly of binary glycosyl phosphodiesters.

#### 3.2. Synthesis of partial structure of galactosamine-modified *Francisella* lipid A and a neoglycoconjugate based thereof

*Francisella* is a highly infectious Gram-negative zoonotic bacterium and the causative agent of tularemia, an extremely contagious lethal pulmonary disease in mammals [124]. Despite clinical and biosecurity importance (*F. tularensis* is classified as a bioterrorism agent [125]), the molecular basis for the pathogenesis of a *F. tularensis* infection remains largely unknown. The major lipid A of *Francisella* has an unusual tetraacylated structure composed of a common β(1→6)-linked diglucosamine backbone which lacks the 4′-phosphate group and the 3′-acyl chain characteristic for enteric lipid A; and contains an α-D-GalN residue that is glycosidically linked to the 1-phosphate group [126]. *Francisella* LPS does not trigger the pro-inflammatory signaling cascade since it cannot be recognised by the TLR4·MD-2 complex owing to the hypoacylated structure of its lipid A and the absence of the 4′-phosphate group [127]. Posttranslational modification of the anomeric phosphate group of lipid A in *Francisella* with α-GalN confers resistance to CAMPs and is associated with augmentation of bacterial virulence [26,128–130]. The full biological consequence of the GalN modification in *Francisella* lipid A is still poorly understood, although it was shown that *F. novicida* mutants which are deficient in GalN modification have attenuated pathogenicity in mice and are capable of stimulating the innate immune response [131].

As a consequence of a unique system of the LPS remodelling enzymes [132–134], *Francisella* produces truncated LPS structure which is composed to 90% from a lipid A portion alone and is not substituted by the core sugars and polymeric O-antigen [126,135]. In this instance, the diglucosamine backbone of *Francisella* lipid A modified by α-D-GalN at the glycosidic phosphate group comprises the antigen-presenting entity of *Francisella* LPS. To assess the antigenic potential of the GalN modification in *Francisella* lipid A, a lipid A-based epitope βGlcN(1→6)-αGlcN(1→P←1)-αGalN **91**, which is conserved in all *Francisella* strains, and a corresponding neoglycoconjugate **92** were synthesised [136]. These compounds could be applied for the generation of diagnostic antibodies or utilized in immunoaffinity assays for detection of *Francisella* infection by direct antigen manifestation in clinical samples [137].

**Scheme 11:**
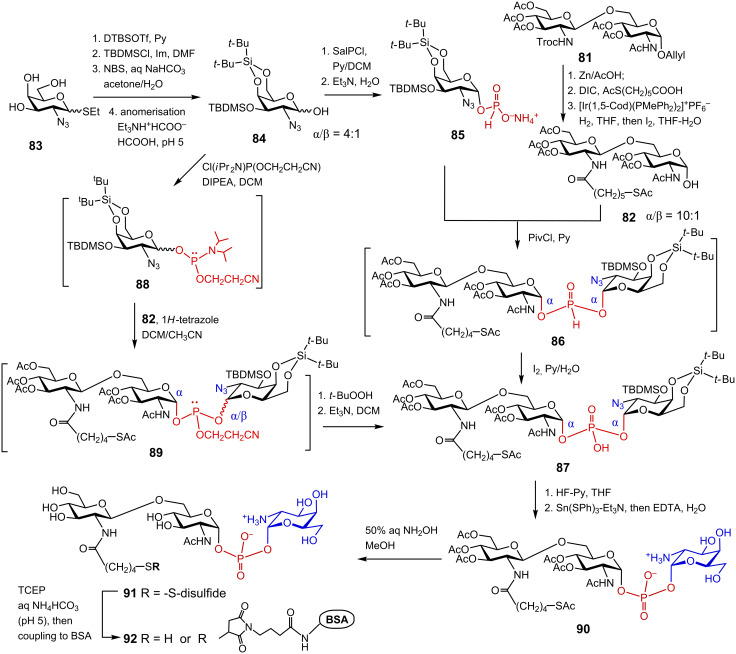
Synthesis of zwitterionic 1,1′-glycosyl phosphodiester: a partial structure of GalN-modified *Francisella* lipid A and a neoglycoconjugate based thereof.

The β(1→6)-linked diglucosamine **81** was prepared by a TMSOTf-assisted glycosylation of the allyl glycoside of the per-acetylated GlcN acceptor having a free 6-OH group by the 2*N*-Troc protected GlcN-based trichloroacetimidate donor [136]. Reductive cleavage of the 2′*N*-Troc protecting group followed by *N,N'*-diisopropylcarbodiimide (DIC)-mediated acylation with 6-thioacetylhexanoic acid afforded a desired β(1→6)-linked disaccharide equipped with a masked spacer group. Cleavage of the 1-*O*-Allyl group by first isomerization to a propenyl group and subsequent aqueous I_2_-mediated hydrolysis provided anomeric α-lactol **82** (α/β = 10:1) entailing an acetyl-protected sulfhydryl-containing spacer (Scheme 11).

For the synthesis of the *Francisella* lipid A backbone having a unique structure which encloses a double glycosyl phosphodiester functionality linking the anomeric centers of two aminosugars, the expediency of the H-phosphonate and phosphoramidite approaches was explored [136]. The synthesis of anomerically pure α-GalN-derived H-phosphonate **85** was performed via regioselective instalment of the 4,6-*O*-*tert*-butylsilylene (DTBS) group into the triol **83**, followed by reaction of the free 3-OH group with TBDMS chloride in the presence of imidazole to furnish a fully protected GalN derivative (Scheme 11). The latter was anomerically deprotected via *N*-bromosuccinimide (NBS)-mediated hydrolysis of the thioethyl glycoside to furnish hemiacetal **84**. The DTBS group exerted a remote α-directing effect [138] which facilitated an enhancement of the α/β ratio in the anomeric lactol **84**. The orthogonally protected GalN hemiacetal **84** (α/β = 3:1) was subjected to phosphitylation reaction with 2-chloro-1,3,2-benzodioxaphosphorin-4-one (salicylchlorophosphite, SalPCl) [139–140]. Since the stereoselectivity of phosphitylation by the P(III)-based reagents commonly reflects the α/β ratio in the starting hemiacetal, the proportion of the α-configured lactol in **84** was additionally enhanced by in situ anomerisation with triethylammonium formate–formic acid buffer (pH 5). The reaction of **84** (α/β = 4:1) with SalPCl in the presence of pyridine afforded glycosyl H-phosphonate **85** which was isolated in pure α-anomeric form as ammonium salt [136]. A pivaloyl chloride (PivCl)-mediated coupling of the H-phosphonate **85** and peracetylated β(1→6) diglucosamine hemiacetal **82** furnished double glycosyl H-phosphonate diester **86**. Oxidation of the intermediate H-phosphonate diester **86** with aqueous I_2_ afforded anomerically pure binary glycosyl phosphodiester **87** entailing αGlcN(1→P←1)αGalN fragment. Application of a nearly pure α-anomeric form of the diglucosamine lactol **82** (α/β = 10:1) and high efficiency of the H-phosphonate coupling allowed for a highly pleasing 85% yield of the glycosyl phosphodiester **87**.

To explore the applicability of the phosphoramidite procedure, the anomeric *N,N*-diisopropyl-2-cyanoethyl phosphoramidite **88** was prepared in situ by treatment of GalN hemiacetal **84** with *N,N*-diisopropyl-2-cyanoethylchlorophosphite in the presence of DIPEA [141]. 1*H*-Tetrazole-mediated coupling of the latter to lactol **82** (α/β = 10:1) afforded a mixture of the intermediate anomeric phosphite triesters **89**. After oxidation with *tert*-butylhydroperoxide and treatment with Et_3_N to remove the cyanoethyl protecting group from the phosphotriester by β-elimination, the target phosphodiester **87** was obtained in a 24% yield. Due to the intrinsic lability of the glycosyl phosphoramidite and glycosyl phosphite intermediates, four sequential transformations were performed as “one-pot” procedure without isolation of individual anomers which ultimately resulted in a poor overall yield.

The progress of a phosphorylation reaction involving phosphorus P(III)-intermediates can be easily monitored by ^31^P NMR spectroscopy. Thus, the H-phosphonate monoester like **85** usually displays a doublet at δ: 4–8 ppm with the coupling constant ^2^*J*_PH_ = 630–650 Hz. After the coupling reaction of the H-phosphonate with the nucleophilic component (hemiacetal **82**), the H-phosphonate diester **86** is expected to have a slightly downfield ^31^P NMR shift δ: 6–12 ppm and a larger coupling constant of ^2^*J*_PH_ = 730–750 Hz. As soon as the H-phosphonate **86** is oxidised to furnish a P(V) phosphodiester **87**, the phosphorus chemical shift usually appears at around δ: 0 ppm. The phosphoramidites like **88** have a characteristic ^31^P NMR chemical shift δ: 150 ppm (two signals corresponding to the *R-* and *S-*diastereomers at phosphorus), whereas the phosphite triesters like **89** display two ^31^P NMR resonances (*R*_p_- and *S*_p_-diastereomers) at δ: 138–142 ppm.

Sequential deprotection of **87** had to be performed under explicitly mild reaction conditions to avoid hydrolysis of the labile double glycosyl phosphodiester functionality. The desilylation of the GalN moiety was accomplished by treatment with diluted HF·Py solution which furnished the corresponding triol. The presence of the terminal thiol precluded application of the Pd-catalysed hydrogenation for the reduction of azido group, so that the Staudinger reaction conditions (using PPh_3_ or PMe_3_) in THF/aq NaOH [142] were initially attempted. The Staudinger reaction did not result in a desired transformation and the alternative procedures for the reduction of azido group were investigated. The best results were achieved upon application of the tin(II) complex [Et_3_NH][Sn(SPh)_3_] [143–144] which quantitatively reduced the 2-azido group in the GalN moiety to yield zwitterionic compound **90**. The use of an excess of the tin(II) reagent caused partial hydrolysis of the GalN fragment in the phosphodiester **90**, unless the tin(II) reagent was trapped by a chelating agent, ethylenediaminetetraacetic acid (EDTA) immediately after the reduction was completed. Final deacetylation was performed under mild basic conditions to afford a zwitterionic phosphodiester **91**. After reduction of the disulfide bond in **91** with tris(2-carboxyethyl)phosphine (TCEP) [145], the resulting thiol was coupled to a maleimide-activated BSA which provided βGlcN(1→6)-αGlcN(1→P←1)-αGalN containing neoglycoconjugate **92**. The epitope can be potentially attached to different surfaces via its thiol-terminated spacer and utilized in diagnostic immuno-assays as capture antigen.

#### 3.3. Synthesis of double glycosyl phosphodiester comprising 4-amino-4-deoxy-β-L-arabinose (β-L-Ara4N) – a partial structure of *Burkholderia* LPS

The *B. cepacia* complex (BCC) is a group of opportunistic bacterial species that can cause lethal pneumonia and septicaemia in patients with cystic fibrosis (CF) and immunocompromised patients resulting in exceptionally high mortality („the cepacia syndrome“) [146]. *Burkholderia* express an unusual lipid A structure which is modified by esterification of the phosphate groups of lipid A by 4-amino-4-deoxy-β-L-arabinose (β-L-Ara4N). A covalent attachment of β-L-Ara4N at the anomeric 1-phosphate group or at the 4’-phosphate group of *Burkholderia* lipid A is estimated as a major pathogenic factor responsible for bacterial virulence and endurance in pulmonary airways [27]. Treatment with antibiotics inflicts selective pressure on BCC in the airways of immunocompromised patients which similarly results in the substitution of the lipid A phosphates by β-L-Ara4N. Addition of the cationic sugar β-L-Ara4N reduces the net negative charge of the bacterial membrane, which enhance bacterial resistance to CAMPs and aminoglycosides [146]. Incidences of profound resistance to polymyxin B – a first choice antibiotic for treatment of multidrug-resistant Gram-negative infections – is also attributed to the β-L-Ara4N modification of the lipid A moiety of LPS [32,147–148]. Accordingly, covalent modification of *Burkholderia* lipid A with Ara4N is crucial for bacterial persistence in the airways of infected patients and results in chronic inflammation and decreased survival [27]. Of special importance are the lipid A structures corresponding to highly pro-inflammatory *B. cenocepacia* [149] and *B. caryophilly* [150] LPS which are modified with β-L-Ara4N exclusively at the glycosidically linked 1-phosphate group of lipid A.

**Scheme 12:**
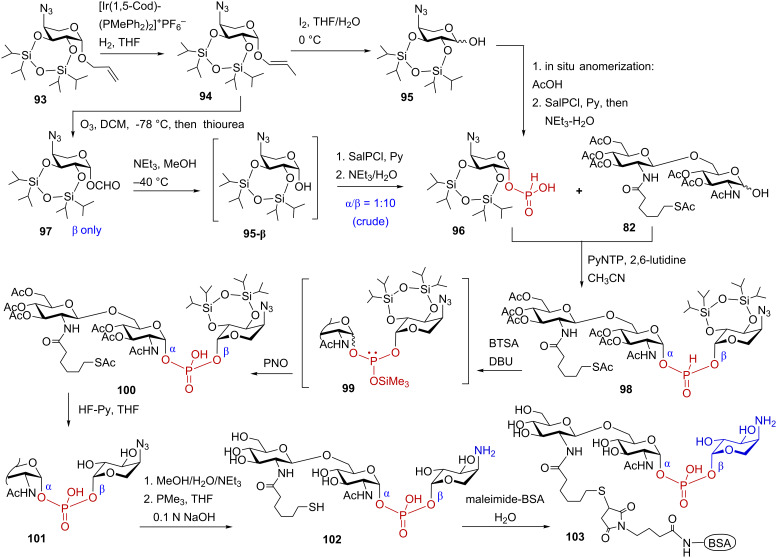
Synthesis of a binary 1,1′-glycosyl phosphodiester: a partial structure of β-L-Ara4N-modified *Burkholderia* Lipid A and a neoglycoconjugate based thereof.

The Ara4N-modified LPS structures can hardly be obtained in pure form by isolation from bacterial cultures owing to intrinsic lability of the glycosyl phosphodiester functionality. The content of β-L-Ara4N in the bacterial isolated is usually reported as “non-stoichiometric” reflecting high degree of heterogeneity of the isolates in respect to substitution of the 1-phosphate group with β-L-Ara4N. To clarify the biological outcome of the Ara4N modification, a reliable synthetic approach toward β-L-Ara4N-containing LPS partial structures was developed [151]. To facilitate the assessment of an immunogenic potential of the unique β-L-Ara4N substitution at the glycosidically linked 1-phosphate group, a neoglycoconjugate **103** entailing an epitope βGlcN(1→6)-αGlcN(1→P←1)-β-L-Ara4N **102** was synthesised in a stereoselective manner [152] (Scheme 12).

For the assembly of binary glycosyl phosphodiester **102**, the synthesis of anomerically pure β-configured H-phosphonate monoester of the orthogonally protected β-L-Ara4N was initially performed (Scheme 12). To this end, the 2,3-*O*-tetraisopropyldisiloxane-1,3-diyl (TIPDS)-protected azide **93** was anomerically deprotected to furnish hemiacetal **95**. Since the stereoselectivity of the phosphitylation at the anomeric center generally relies on the anomeric ratio in the lactol precursor [153–154], the preparation of anomerically enriched hemiacetals which can be straightforwardly converted into the corresponding H-phosphonates comprised the foremost synthetic challenge. When the cleavage of the anomeric allyl group was carried out by sequential double bond isomerisation with [Ir(1,5-Cod)(PMePh_2_)_2_]^+^PF_6_^−^ to give propenyl glycoside **94**, followed by I_2_-assisted prop-1-enyl cleavage, an anomeric mixture **95** (α/β = 1:1) was obtained. Lactol **95** could be enriched with the β-anomer (α/β = 1:3) by treatment with CHCl_3_/MeOH/AcOH solution. Subsequent phosphitylation by reaction with salicylchlorophosphite (SalPCl) [139] in pyridine gave rise to the anomeric H-phosphonates (α/β = 1:3), whereas the β-anomer **96** could be isolated in a moderate 35% yield.

To achieve a better stereoselectivity, a novel procedure for traceless removal of the allyl group in β-allyl glycoside **93** without affecting the axial anomeric configuration at C-1 was elaborated. After allyl group isomerization, the anomeric prop-1-enyl ether **94** was oxidised by ozonolysis to give a stable formyl intermediate **97** under mild conditions (Scheme 12) [155–157]. The formate group was hydrolysed by methanolysis (NEt_3_, MeOH, −40 °C) to furnished solely β-configured lactol **95β** and volatile methyl formate, so that the crude β-lactol could be directly subjected to phosphitylation without a need of chromatographic purification (which would result in a rapid anomerisation). A predominant formation of the β-configured H-phosphonate **96** was achieved by application of highly reactive phosphitylating reagent SalPCl, which quickly trapped the excess of axial β-lactol in **95β**, such that the initial α/β ratio was preserved and the anomerically pure β-glycosyl H-phosphonate **96** was obtained in 78% yield. Glycosyl-H-phosphonate **96** was initially coupled to the β(1→6)–linked diglucosamine lactol **82** [136] using pivaloyl chloride (PivCl) as activating agent [153–154,158] to furnish H-phosphonate glycosyl phosphodiester **98** as an anomeric mixture at GlcN moiety. Oxidation of **98** by treatment with aqueous I_2_ at −40 °C afforded anomerically pure binary glycosyl phosphodiester **100**, whereas the more labile β-anomeric product was destroyed upon aqueous I_2_-mediated oxidation and isolation of the phosphodiester **100** by chromatography on silica gel [159]. Since the PivCl-mediated H-phosphonate coupling can be often accompanied by concomitant side-reactions (formation of P-acyl byproducts [140] resulting from an over-reaction of **96** or **98** with PivCl or formation of GlcNAc-derived oxazolines in the presence of an excess of chloroanhydride) [141], phosphonium type coupling reagents were optionally explored. Accordingly, the H-phosphonate **96** was activated by 3-nitro-1,2,4-triazol-1-yl-tris(pyrrolidin-1-yl)phosphonium hexafluorophosphate (PyNTP), which selectively reacted with the electrophilic phosphorus atom of the H-phosphonate to form a P–N activated intermediate [160–161]. The later was smoothly coupled to the nucleophilic component, the hemiacetal **82**. To circumvent possible hydrolysis of the binary glycosyl H-phosphonate diester **98** during the aqueous I_2_-mediated oxidation step, the oxidation was performed in anhydrous conditions by transforming the tetra-coordinated H-phosphonate **98** into the three-coordinated silyl phosphite **99** (via treatment with *N,O*-bis(trimethylsilyl)acetamide (BTSA) in the presence of DBU) [162] followed by oxidation of **99** with 2-(phenylsulfonyl)-3-(3-nitrophenyl)oxaziridine (PNO) to furnish 1,1’-glycosyl phosphodiester **100**. The stepwise deprotection of **100** included a treatment with HF·Py to remove the TIPDS protecting group, a deacetylation of **101** (including deprotection of the 6-thioacetylhexanoyl residue) with MeOH/H_2_O/NEt_3_ and a final reduction of the 4-azido group by reaction with trimethylphosphine [142] in aq NaOH/THF which provided **102**. The formation of a disulfide bond was inhibited by application of reducing agent (PMe_3_), so that the trisaccharide **102** could be directly coupled to a maleimide-activated BSA via a sulfhydryl-containing spacer group to furnish the neoglycoconjugate **103**. Thus, a novel efficient approach for anomeric deallylation with retention of configuration allowed for the stereoselective synthesis of anomerically pure β-L-Ara4N glycosyl H-phosphonate and β-L-Ara4N-containing antigenic LPS epitope as useful biochemical probe and potential diagnostic agent.

#### 3.4. Synthesis of *Burkholderia* lipid A modified with glycosyl phosphodiester-linked β-L-Ara4N

The pro-inflammatory activity of *Burkholderia* LPS isolates, which belongs to the major virulence factors of BCC species, has been extensively studied. Heterogeneous tetra- and pentaacylated LPS/lipid A from *B. mallei* [163], *B. multivorans* [164], *B. cenocepacia* [149,165], *B. cepacia* [27] and *B. dolosa* [166] were determined as potent stimulators of the TLR4·MD-2-mediated cellular responses. Though it is generally believed that only hexaacyl lipid A (such as from *E. coli*) is capable of interacting with TLR4 complex and eliciting powerful innate immune response [18,167], underacylated β-L-Ara4N modified *Burkholderia* LPS isolates induced the expression of pro-inflammatory cytokines in vitro, and the efficiency of cytokine production was comparable with that induced by hexaacylated *E. coli* LPS [149]. The intrinsic lability of the aminosugar modification of the glycosyl phosphate group of lipid A results in a high degree of heterogeneity of lipid A preparations obtained from *Burkholderia* isolates in respect to the degree of β-L-Ara4N substitution which is commonly indicated as “non-stoichiometric”. The lipid chain content in *Burkholderia* LPS also varies from species to species which makes it difficult to determine the structural characteristics of *Burkholderia* lipid A accountable for its unusual immuno-stimulating activity [168–169]. Since the 1-phosphate group of lipid A is directly involved in the formation of the dimeric MD-2·TLR4-LPS complex [42], the appendage of β-L-Ara4N might enhance the efficiency of dimerization via ionic attraction. In order to elucidate the structural determinants responsible for the unique pro-inflammatory potential of *Burkholderia* lipid A, the pentaacylated *Burkholderia* lipid A esterified by β-L-Ara4N at the anomeric phosphate **101** and its Ara4N-free counterpart **102** corresponding to native *Burkholderia* LPS were chemically synthesised [161].

**Scheme 13:**
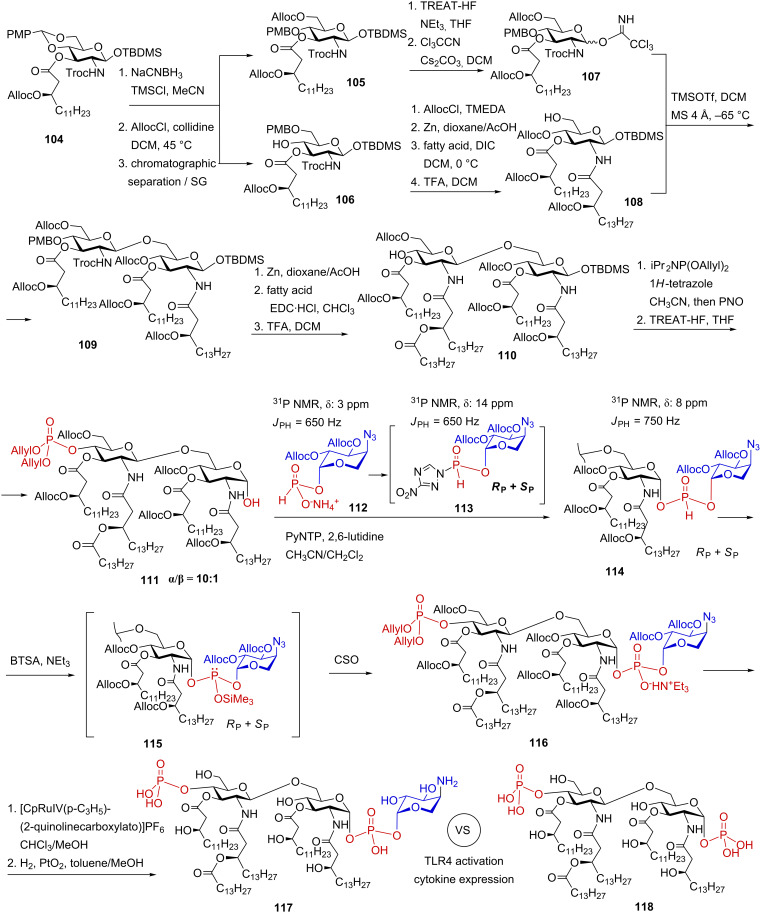
Synthesis of *Burkholderia* lipid A containing binary glycosyl phosphodiester linked β-L-Ara4N.

The synthesis of fully orthogonally protected tetraacylated βGlcN(1→6)GlcN intermediate **109** commenced with the preparation of the GlcN-based *N*-Troc protected imidate donor **107** and the GlcN-derived bis-acylated 6-OH acceptor **108** (Scheme 13). Reductive opening of the *p*-methoxybenzylidene acetal protecting group in **104** with sodium cyanoborohydride and trimethylsilyl chloride in acetonitrile furnished a mixture of 6-OH and 4-OH (compound **106**) co-migrating regioisomers. This inseparable mixture was subjected to regioselective 6-*O*-protection with allyloxycarbonyl group by the action of allyloxycarbonyl chloride in the presence of *sym-*collidine, which transformed the 6-OH regioisomer into the 6-*O*-Alloc protected derivative **105**, whereas **106** having less reactive secondary 4-OH group did not react with AllocCl in the presence of a mild base. The resulting mixture – 6-*O*-Alloc-4-*O*-PMB protected **105** and 6-*O*-PMB protected **106** – was readily separated by conventional chromatography on silica gel. The anomeric TBDMS group in **105** was cleaved by treatment with triethylamine tris(hydrogenfluoride) (TREAT-HF) buffered by Et_3_N (pH 6.5) which kept the acid labile 6-*O*-*p*-methoxybenzyl (PMB) group unaffected. The resultant hemiacetal was converted into fully protected trichloroacetimidate donor **107**.

The free secondary 4-OH group in **106** was protected by reaction with AllocCl in the presence of the stronger base *N,N,N',N'*-tetramethylethylendiamine (TMEDA) [170]. The *N*-Troc group was subsequently reductively cleaved by treatment with Zn in acetic acid/dioxane followed by acylation of the intermediate amine by DIC-activated (*R*)-3-(allyloxycarbonyloxy)hexadecanoic acid. Succeeding acidic hydrolysis of the PMB group with trifluoroacetic acid furnished the 6-OH acceptor **108**. A TMSOTf–promoted glycosylation of **108** by the imidate donor **107** furnished a tetraacylated β(1→6)-linked disaccharide **109** (Scheme 13). Reduction of the 2´-*N*-Troc group by use of Zn in AcOH followed by *N*-acylation with (*R*)-3-acyloxyalkanoyl fatty acid in the presence of 1-ethyl-3-(3-dimethylaminopropyl) carbodiimide hydrochloride (EDC∙HCl) gave fully protected pentaacylated intermediate which was treated with TFA in CH_2_Cl_2_ to promote hydrolysis of 4’-*O*-PMB group to furnish **110**. Compound **110** was phosphitylated at O-4’ by reaction with diallyl(*N,N-*diisopropyl)phosphoramidite [171] in the presence of 1*H*-tetrazole and successive oxidation of the intermediate phosphite triester with PNO [172] to provide protected 4‘-*O*-phosphate. The anomeric 1-*O*-TBDMS group in the latter was removed by treatment with TREAT-HF to give hemiacetal **111**. Since lactol **111** had to be stereoselectively coupled to the Ara4N H-phosphonate **112**, the anomeric preference of the α-configured lactol was especially important. Stabilization of the axial orientation of the 1-OH in **111** via intramolecular hydrogen bonding with the 2-NH group [154] ensured high proportion of the α-configured lactol (α/β = 10:1) and improved stereoselectivity in the next coupling step. Anomerically pure 2,3-di-*O*-Alloc protected β-L-Ara4N glycosyl H-phosphonate **112** was synthesised starting from 1-*O*-Allyl-4-azido β-L-Ara4N [173] in four steps [161].

The coupling of lactol **111** to the β-L-Ara4N glycosyl H-phosphonate **112** was promoted by 3-nitro-1,2,4-triazol-1-yl-tris(pyrrolidin-1-yl)phosphonium hexafluorophosphate (PyNTP) in the presence of 2,6-lutidine and afforded binary glycosyl H-phosphonate diester **114**. The H-phosphonate coupling reaction proceeded through formation of the tetracoordinated P(III) intermediates: H-pyrophosphonates [174] and nitrotriazol-1-yl-phosphites [175], such as β-L-Ara4N-nitrotriazol-1-yl-H-phosphonate **113** (^31^P NMR (δ): 13 and 14 ppm, *J*_PH_ = 650 Hz), which instantly reacted with α-hemiacetal **111**. ^31^P NMR spectroscopy was used to confirm the formation of a labile intermediate H-phosphonate diester **114** which displayed representative PH-coupled signals conforming with the formation of *R* and *S* diastereomers at phosphorus (^31^P NMR (δ): 7.6 and 8.0 ppm, *J*_PH_ = 750 Hz). Due to exceptional lability of the binary glycosyl H-phosphonate diester **114**, the oxidation could not be performed under standard H-phosphonate chemistry conditions (aq. iodine) and, therefore, was accomplished in anhydrous conditions. To this end, the tetra-coordinated H-phosphonate was transformed into the three-coordinated phosphite **115** by reaction with *N,O*-bis(trimethylsilyl)acetamide [162,176] in the presence of Et_3_N. The reaction was monitored by ^31^P spectroscopy which confirmed the formation of the intermediate phosphite **115**. Subsequent oxidation with (1*S*)-(+)-(10-camphorsulfonyl)oxaziridine (CSO) [177] furnished P(V) 1,1´-glycosyl phosphodiester **116**. Total cleavage of the Alloc- and Allyl- protecting groups in **116** was performed under mild neutral conditions [178] by treatment with [CpRu(IV)(π-C_3_H_5_)(2-quinolinecarboxylato)]PF_6_ complex [179–180], so that a labile double glycosyl phosphodiester linkage was not affected. Finally, the azido group was reduced by hydrogenation on PtO_2_ to give the target β-L-Ara4N-modified *Burkholderia* lipid A **117**. The availability of homogenous structurally defined synthetic β-L-Ara4N-modified *Burkholderia* lipid A provided a reliable tool for immunobiological studies. The immunomodulating potential of synthetic β-L-Ara4N-modified *Burkholderia* lipid A **117** and its non-modified synthetically prepared counterpart **118** was assessed in TLR4-transfected human embryonic kidney HEK293 cells by monitoring the activation of NF-κB signaling and in the human monocytic macrophage cell line THP-1. The β-L-Ara4N-modified lipid A **117** was considerably less efficient than *E. coli Re*-LPS in triggering the NF-κB signaling, however, it induced the expression of significantly higher levels of IL-8 compared to the non-modified pentaacyl bisphosphate lipid A **118** which was inactive at wide concentration range. Thus, the chemical synthesis of β-L-Ara4N-modified lipid A helped to reveal its immuno-modulatory potential and to demonstrate an enhancement of the pro-inflammatory activity of *Burkholderia* lipid A esterified by β-L-Ara4N at the glycosidically-linked phosphate group.

## Conclusion

The synthesis of carbohydrate-based biomolecules is an area of fundamental and practical importance. Owing to immunomodulating capacities of lipid A and related glycolipids, the development of facile synthetic strategies toward these complex glycoconjugates have received particular attention. Despite huge progress achieved in the preparation of lipid A by combinatorial bioengineering of LPS and improved isolation techniques, the chemical synthesis remains the only source for sufficient amounts of structurally well-defined homogeneous materials which are completely free from any potentially pro-inflammatory biological contaminations and are suitable for biomedical or diagnostic application. Moreover, the intrinsic instability of particularly complex lipid A variants such as aminosugar-modified lipid A, renders the chemical synthesis to a single option for obtaining structurally integral compounds for biological studies. The inherent hybrid molecular structure of lipid A combining sugar-derived phosphorylated polar head group and multiple lipid moieties poses additional challenges to elaboration of efficient synthetic methodologies. Newly developed strategies allowed for divergent synthesis of LPS partial structures entailing lipid A that varies in the acylation pattern and the number of phosphate groups by the use of a single orthogonally protected disaccharide precursor. Application of advanced P(III) chemistry aided the development of stereoselective synthesis of binary glycosyl phosphodiesters comprising two aminosugars.
